# Adeno-Associated Virus Gene Therapy Development: Early Planning and Regulatory Considerations to Advance the Platform Vector Gene Therapy Program

**DOI:** 10.1089/hum.2024.230

**Published:** 2025-03-06

**Authors:** Richa Madan Lomash, Jean Dehdashti, Oleg A. Shchelochkov, Randy J. Chandler, Lina Li, Irini Manoli, Jennifer L. Sloan, Pramod Terse, Xin Xu, Dimah Saade, Rodica Stan, PaVe-GT team, Gilberto V. Averion, Krishna Balakrishnan, Steven J. Burden, Eggerton Campbell, Catherine Chen, Eun-Young Choi, Claire Driscoll, Dukhanina Oksana, Susan Ferry, A. Reghan Foley, Janelle Geist Hauserman, Venkata Mangalampalli, Christopher Mendoza, Asvelt Nduwumwami, Julien Oury, Forbes D. Porter, Deanna Portero, Lili Portilla, Jachinta Rooney, Mitali Tambe, Joshua Todd, London Toney, Carol Van Ryzin, Sury Vepa, Erik Wagner, Amy Wang, Yaqun Zou, Philip J. Brooks, Donald C. Lo, Carsten G. Bönnemann, Charles P. Venditti, Anne R. Pariser, Elizabeth A. Ottinger

**Affiliations:** ^1^ Therapeutic Development Branch, Division of Preclinical Innovation, National Center for Advancing Translational Sciences (NCATS), NIH, Rockville, Maryland, USA; ^2^ Division of Rare Diseases Research and Innovation, NCATS, NIH, Rockville, Maryland, USA; ^3^ Organic Acid Research Section, Metabolic Medicine Branch, National Human Genome Research Institute (NHGRI), NIH, Bethesda, Maryland, USA; ^4^ Neuromuscular and Neurogenetic Disorders of Childhood Section, National Institute of Neurological Disorders and Stroke (NINDS), NIH, Bethesda, Maryland, USA.

**Keywords:** rare diseases, target product profile, INTERACT meeting, gene therapy, PaVe-GT, propionic acidemia, PCCA, AAV9

## Abstract

Gene therapy development presents multiple challenges, and early planning is vital in the successful implementation of such programs. The Platform Vector Gene Therapy (PaVe-GT) program is a National Institutes of Health (NIH) initiative developing adeno-associated virus (AAV) gene therapies for four low-prevalence rare diseases. Utilizing the platform-based approach, the program aims to incorporate efficiencies throughout the preclinical and clinical development processes followed by public dissemination of scientific and regulatory learnings. Early in development, the establishment of a Target Product Profile (TPP) by the research team is a critical step to guide product development and align preclinical studies to clinical objectives. Based on the specific needs of the investigational product as defined in the TPP, an overall regulatory strategy can then be outlined to meet the regulatory requirements for the first-in-human clinical trials. During the preclinical phase of development, sponsors may request meetings with the Food and Drug Administration (FDA) to gather feedback on the planned studies and regulatory strategy. To pave the way for PaVe-GT’s first investigational AAV gene therapy lead candidate, AAV9-hPCCA, we sought early feedback from the FDA utilizing an INitial Targeted Engagement for Regulatory Advice on CBER/CDER ProducTs (INTERACT) meeting. Here, we elaborate on the value of establishing a TPP and the FDA INTERACT meeting by including our initial AAV9-hPCCA TPP, detailing our INTERACT meeting experience, providing all corresponding regulatory documentation, and highlighting lessons learned. The regulatory documents along with templates developed by our program can also be found on the PaVe-GT website (https://pave-gt.ncats.nih.gov/). This communication aims to provide stakeholders with resources that can be applied to drug development programs in establishing a viable regulatory path to clinical trial initiation.

## INTRODUCTION

Gene therapy development is an advancing field with wide applicability for rare diseases, the majority of which are monogenic.^1–3^ The number of preclinical and clinical gene therapy programs has increased substantially in recent years with 2070 gene therapies in the biopharmaceutical industry pipeline of which 24% are adeno-associated virus (AAV) gene therapies.^4,5^ To date, there have been AAV gene therapy approvals for eight indications globally.^6,7^ Despite advancements, the development of gene therapies continues to present challenges in access to expertise and resources, high costs, product manufacturing, and navigation of numerous regulatory requirements.^8,9^

**Figure 1.**
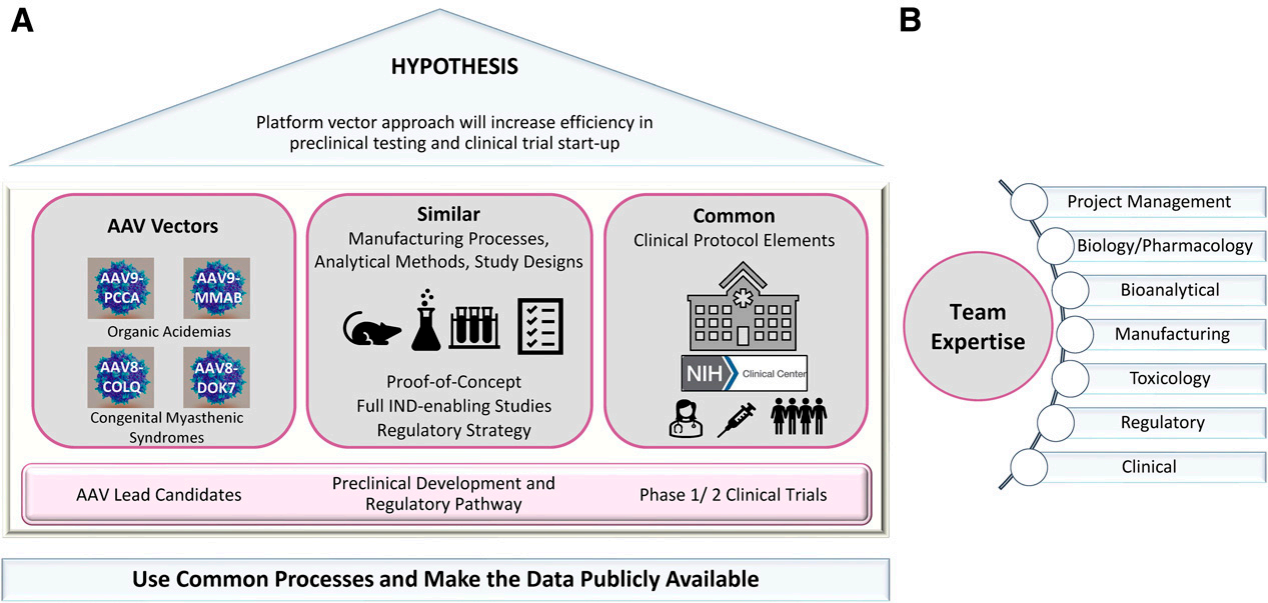
Platform Vector Gene Therapy (PaVe-GT) approach. **(A)** To test the PaVe-GT hypothesis, our approach includes utilizing AAV as a platform vector, similar preclinical development processes, study designs, regulatory pathway and common clinical protocol elements, wherever possible. The diseases under study in the PaVe-GT platform include four rare diseases. The two organic acidemias under study are propionic acidemia (caused by PCCA deficiency) and isolated methylmalonic acidemia (MMAB deficiency/cobalamin type B methylmalonic acidemia). The two congenital myasthenic syndromes under study are COLQ deficiency and DOK7 deficiency. The outcomes of the program are being publicly shared on the PaVe-GT website. **(B)** The PaVe-GT team is comprised of basic researchers, disease and drug development experts supported by a strong project management team as depicted in the figure. All disciplines work in coordination, and the coordinated efforts of all team members are instrumental for the success of the program.

The National Institutes of Health (NIH) embarked upon a gene therapy program called Platform Vector Gene Therapy (PaVe-GT)^10^ to test the hypothesis whether a platform approach can increase efficiencies in preclinical studies and clinical development. While implementing this program, the PaVe-GT team is committed to public dissemination of all learnings^10^ (Fig. 1A). AAV has been selected as a platform vector, and a multidisciplinary team has been assembled (Fig. 1B) to strategize and execute the drug development plans for four rare diseases—two organic acidemias and two neuromuscular disorders. The team aims to incorporate learnings moving from one product to the next, following an integrated approach throughout preclinical and clinical development using similar processes, study designs, and regulatory pathway. The scientific and regulatory knowledge gained during the program’s lifecycle and the processes adopted will be publicly disseminated to inform and enable other gene therapy development programs.

The focus of this communication is to help readers understand the significance of early planning during the drug development process utilizing PaVe-GT’s first investigational gene therapy candidate, AAV9 human Propionyl-CoA Carboxylase, alpha subunit (AAV9-hPCCA) or National Center for Advancing Translational Sciences (NCATS)-BL0746, as a case example. This paper emphasizes the value of establishing a Target Product Profile (TPP) and having early interactions with the Food and Drug Administration (FDA), specifically the INitial Targeted Engagement for Regulatory Advice on Center for Biologics Evaluation and Research (CBER)/Center for Drug Evaluation and Research (CDER) ProducTs (INTERACT) meeting. The AAV9-hPCCA TPP, complete INTERACT package along with FDA feedback is included in the supplemental information, and the template used in drafting these documents is posted on the PaVe-GT website.

## BACKGROUND

The drug development process is complex, lengthy and costly, and comes with the uncertainty of whether the lead investigational candidate will be safe and effective in patients. Reasons for failure can range from scientific and strategic to operational and commercial issues.^11^ Scientific challenges include demonstrating efficacy in appropriate disease models, development of assays, complex manufacturing processes, and toxicology assessment.^11–13^ For rare diseases, clinical trial design and endpoint selection are difficult due to poor understanding of disease biology, phenotypic heterogeneity,^14^ lack of validated biomarkers, incomplete understanding of disease natural history, and small populations that often include pediatric patients^14–16^ With nearly 70% of rare diseases starting in childhood,^15^ it can be challenging to establish proof of direct benefit in animals and appropriately design safety studies in support of the pediatric population. Furthermore, an insufficient understanding of how preclinical studies translate toward a first-in-human (FIH) trial in accordance with regulatory requirements may result in roadblocks at various stages of the drug development process. Based on our experience, failure to incorporate regulatory planning early enough may lead to delays in advancing a lead candidate to clinical testing. Potential regulatory hurdles can arise from inadequate use of publicly available FDA resources and precedence, submission of incomplete meeting packages to regulatory authorities with respect to supporting scientific rationale and data, and underutilization of FDA meetings and interactions throughout product development. Operational delays range from funding and timeline deviations to supply chain issues and intellectual property challenges.^13,17^

To maximize a program’s success, it is critical to incorporate scientific expertise, project management and effective risk mitigation strategies throughout the project lifecycle. Ensuring alignment of study outcomes and their timing with the regulatory considerations is important in the planning and execution of Investigational New Drug (IND) enabling studies during preclinical development.

### Target product profile (TPP)

Per the World Health Organization (WHO) definition, A ‘Target Product Profile’ (TPP) outlines the desired ‘profile’ or characteristic of a target product that is aimed at a particular disease or diseases.^18^ It is used as a common project management and strategic tool to guide product development^19,20^ and facilitate regulatory submissions and communications. A TPP is created with consideration of the product labeling as preclinical and clinical programs develop.

**Figure 2.**
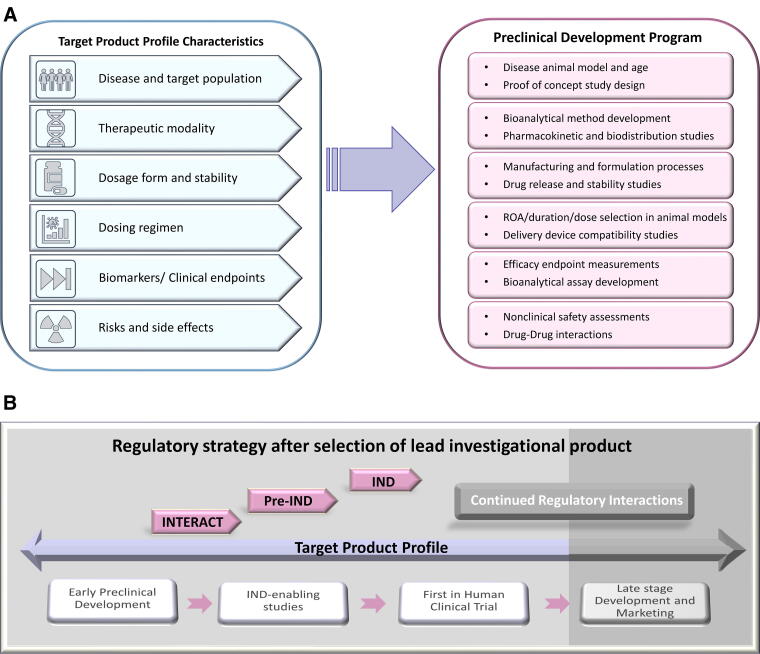
Regulatory milestones and TPP. **(A)** This figure highlights how each component of a TPP can inform various aspects of the preclinical development program and assist in the study design and implementation to maximize program success and shorten the timeline. ROA: Route of administration. **(B)** After selection of a lead candidate, early establishment of a TPP as a guiding tool, in combination with stage appropriate regulatory interactions, can be instrumental for successfully moving a program through the various phases of development. Regulatory interactions can be initiated during early product development and continue until the IND submission, which is a significant milestone in the process and allows for the initiation of the FIH trial. Late-stage product development also entails the TPP and continued regulatory interactions (Note: late-stages not discussed in the article).

As illustrated in Figure 2A, a TPP consists of the product’s characteristics and attributes, such as the disease indication, target population, safety and tolerability information, dosage form and frequency, drug stability information, dosing regimen and clinical efficacy endpoints. Components of a TPP are important to inform study parameters in preclinical development, including but not limited to demonstrating proof of direct benefit in animal models, non-clinical toxicology assessments, assay development, and the manufacturing process. Incorporating clinical considerations for a lead candidate in the TPP early on better informs the design of IND-enabling studies to streamline the preclinical drug development process.^21^ TPP is a living document where scientific and regulatory aspects merge to drive programmatic decisions, and it can be continually updated through the project lifecycle. We have included the initial TPP for AAV9-hPCCA as Supplementary Data S1. Additional resources for creating a TPP are available.^22–25^

### Regulatory considerations: Strategy and interactions

An understanding of the available resources including guidance documents and potential meetings with regulatory agencies throughout different stages of development is crucial for advancing programs efficiently. Meetings with the FDA can be utilized for gaining feedback and concurrence on preclinical studies used in support of an IND application and the initiation of a clinical trial. Early interactions also help initiate a relationship between the sponsor and the FDA review team. It is important to note that the goal of both the sponsor and the FDA is to advance the development of a lead candidate for clinical testing to improve public health. During the preclinical phase of development, sponsors have the option to request different meetings with the agency (*e.g.,* INTERACT and pre-IND) (Fig. 2B).^26^ Engaging with the FDA early during product development enables the sponsor to implement feedback for any given area (*e.g.,* preclinical models, manufacturing, toxicology studies, clinical protocol synopsis, etc.) and pivot where needed to ensure that the development program meets regulatory requirements.

## REGULATORY STRATEGY FOR AAV9-HPCCA

**Figure 3.**
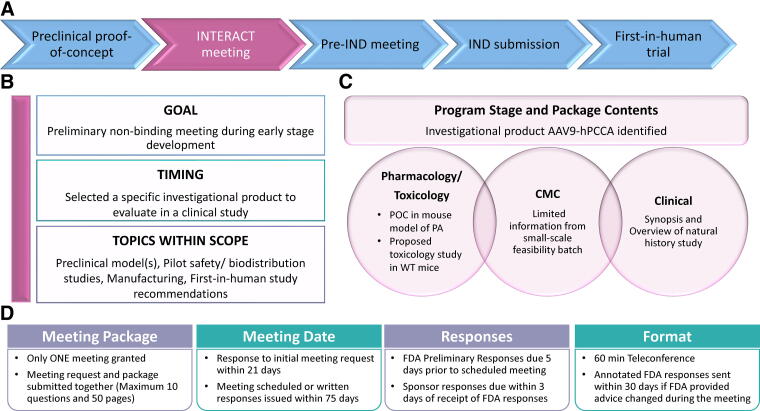
Regulatory strategy for AAV9-hPCCA. **(A)** AAV9-hPCCA regulatory strategy is outlined. The first milestone was an INTERACT meeting and the feedback helped us move the program forward and prepare for a subsequent pre-IND meeting. For this program, FIH trials will commence after (FDA)’s review of our IND (with receipt of an FDA safe-to-proceed) and completion of other necessary clinical steps. **(B)** INTERACT meeting goals, timing and topics within scope are specified. **(C)** AAV9-hPCCA INTERACT package contents included *in vivo* POC study results, IND enabling Good Laboratory Practice (GLP) toxicology study plan, CMC, and clinical information. **(D)** Logistical information related to the procedures and timelines for an INTERACT meeting.

Upon completion of the preclinical proof-of-concept (POC) studies for AAV9-hPCCA,^27^ we utilized the FDA INTERACT meeting to get advice and gain concurrence on our development plan. The overall program strategy is outlined in Figure 3A, and the following sections focus on the team’s INTERACT experience and takeaways for AAV9-hPCCA.

### INTERACT meeting

As stated in FDA SOPP 8101.1,^28^ to qualify for an INTERACT meeting, a sponsor must have selected a lead investigational candidate to evaluate in a clinical study since all questions and clarifications stem from this product. Qualified sponsors should utilize an INTERACT meeting early in preclinical development, after completion (or near-completion) of POC studies with their lead candidate. Discussion topics during INTERACT include early product characterization and manufacturing process; POC studies and preclinical models; pilot safety, biodistribution studies, and FIH study recommendations (desired patient population age, route of administration, novel delivery devices, etc.) (Fig. 3B).^28^ To help understand the scope of INTERACT meetings, the FDA has provided example questions and topics as guidance for what is (or is not) appropriate for this meeting type.^28^ Failure to comply with the requirements may lead to denial of an INTERACT meeting. The reasons for denial vary from a package being deficient or project stage being too premature or advanced.^29^ A request may be too advanced for an INTERACT meeting and more appropriate for a pre-IND meeting if, for example, a sponsor has completed some safety studies and is at the point of design and conduct of definitive toxicology studies. The FDA website contains more information on common reasons for denying INTERACT meetings.^29^

### Contents of the AAV9-hPCCA INTERACT meeting request and package

The information included in an INTERACT package may vary for each program depending on the stage of development. An INTERACT package includes administrative information, lead investigational candidate details, disease background, and a summary of the development plan (including non-clinical, chemistry, manufacturing and controls [CMC] information, clinical synopsis, and regulatory pathway). The questions included in the package for feedback should be presented with appropriate technical background and supportive scientific data. For AAV9-hPCCA, we had conducted preliminary POC studies and initiated CMC process development. The complete AAV9-hPCCA INTERACT meeting request and briefing package can be found in Supplementary Data S2. The AAV9-hPCCA INTERACT package summary and description of the program stage is detailed below and in Figure 3C.

We included:

1. ***In vivo* POC studies:** As a measure of efficacy, we presented results from *Pcca* knockout animals showing improved survival and reduced levels of the characteristic metabolite, plasma 2-methylcitrate, after treatment with AAV9-hPCCA. We also included transgene mRNA and protein expression results from tissues of relevance.
2. **IND enabling GLP toxicology study plan:** We included the proposed study design, dosing regimen and planned measurements.
3. **Clinical information:** Disease manifestations of *PCCA-*related PA, with particular emphasis on the selected pediatric patient population, ongoing PA natural history study,^30–32^ FIH clinical protocol synopsis with study design and endpoints were presented.
4. **CMC information**: Information from the small-scale feasibility batch was included to receive initial guidance on further process development and manufacturing.
5. **PaVe-GT Platform:** We detailed the platform approach and discussed the possibility of leveraging data across the four gene therapy products.

Based on the information available for our program, questions were formulated in a manner that enabled gathering advice specific to AAV9-hPCCA in different technical areas along with PaVe-GT platform-associated questions. Unlike some other meeting types, the INTERACT meeting request and briefing package are submitted concurrently.^28,29^ (Fig. 3D).

### Compilation and submission of the AAV9-hPCCA INTERACT meeting request and package

**Figure 4.**
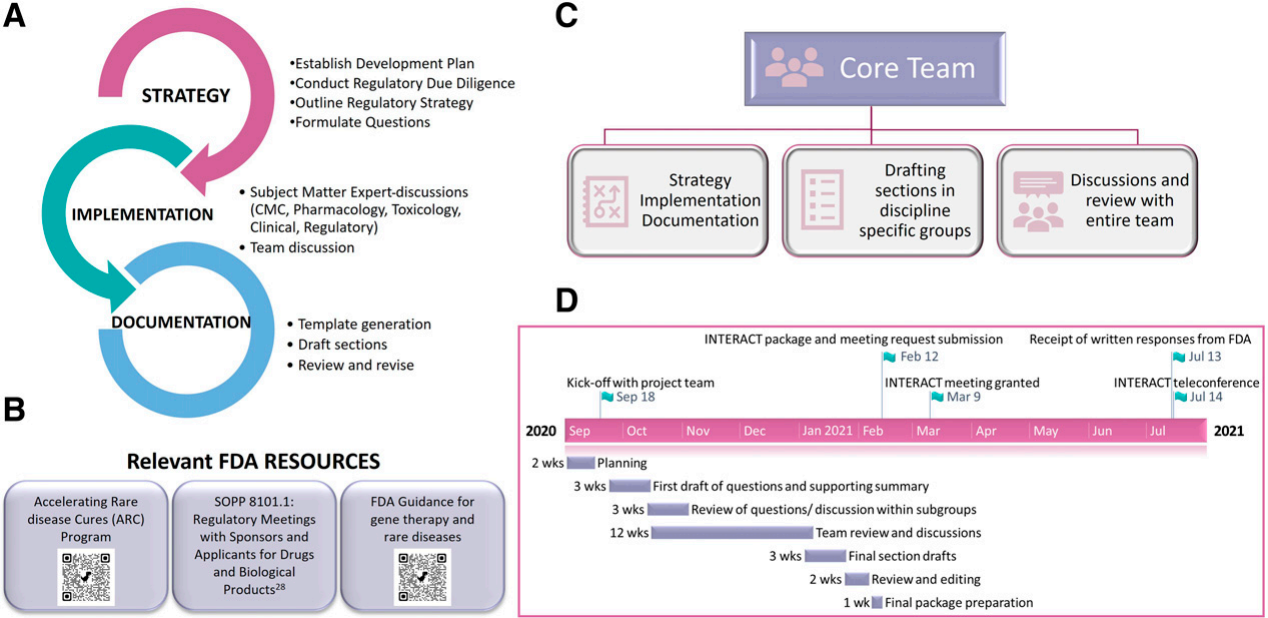
Approach utilized by the PaVe-GT team for the INTERACT package preparation. **(A)** Our process for compilation of the package can be divided into three stages: strategy, implementation, and documentation. **(B)** FDA resources valuable for INTERACT package preparation and defining the drug development plan are included along with hyperlinks. **(C)** The core project management team led the drafting and package preparation working closely with the technical leads, regulatory experts, and clinicians. **(D)** Steps and timeline of AAV9-hPCCA INTERACT package preparation: Upon initial due diligence and planning, we held a kick-off meeting to inform all team members of the scope and content of an INTERACT meeting request and package. Subsequently, we discussed in smaller and larger groups to generate an initial draft of questions along with a supporting summary. Open dialogue and discussions were needed to solidify the development plan and finalize section drafts. The final stages included reviewing the complete package, editing, and formatting.

We drafted the INTERACT package in-house, and to help other groups, we have detailed our approach below. As outlined in Figure 4A, we established the drug development plan, conducted due diligence on available information, and outlined the regulatory steps. This strategy helped us to understand program-specific challenges and accordingly formulate focused questions requiring feedback. Refining the drug development plan was an iterative process, and outlining the studies to support the planned clinical trial required broad discussions and scenario analyses. Discussions with individual subject matter experts (SMEs) across disciplines (*e.g.,* Biology, CMC, Toxicology, Clinical, and Regulatory) helped incorporate granular details followed by input from the larger team, to generate a comprehensive package. We started adding information into our INTERACT template^33^ to produce an initial draft that went through several rounds of review before finalizing. We utilized FDA guidance^34^ and resources for developing the AAV9-hPCCA drug development plan and compilation of our package (Fig. 4B). A core project management team led the overall effort by managing all communications, driving discussions and drafting the package (Fig. 4C). Working together with the technical leads, the core team was instrumental in defining the overall development plan and gathering information for the package. This team ensured that proposed studies were aligned with the TPP characteristics, and that package preparation was achieved efficiently. The complete process for compiling and submitting the AAV9-hPCCA package took approximately 5 months, and the steps are listed in Figure 4D. Following the submission, the FDA acknowledged receipt of the INTERACT package and provided a meeting date.

After the INTERACT meeting for AAV9-hPCCA in 2021, there have been a few changes to the FDA process, which are selectively highlighted.^26,28,29^ Per the 2023 Prescription Drug User Fee Act (PDUFA) reauthorization performance goals and procedures, INTERACT meeting confirmation or denial will now be issued within 21 calendar days of FDA’s receipt of the meeting request and package, and the meeting will be scheduled within 75 calendar days. The FDA also informs the sponsor if reviewers from specific discipline areas are unable to participate in the meeting.^28^ The FDA sends preliminary responses to the sponsor, and if, based on the teleconference discussion, the FDA advice changes, the written responses will be annotated and resent (Fig. 3D). For sponsors requesting written responses only, the initial written responses will serve as the final FDA meeting minutes.

### FDA review and INTERACT meeting preparation

To accommodate for the short timeframe between receiving FDA preliminary comments and for the sponsor to provide a response to FDA with selected topics for discussion, proactive preparation is critical. We initially held internal meetings with SMEs to discuss potential responses from the FDA and our responses. After receiving FDA preliminary comments, we selected the topics most critical to the program to maximize the benefit from the scheduled meeting. For example, based on FDA preliminary comments, the team determined the need to elaborate on the natural history and disease progression in the pediatric and adult patient populations. We prepared slides highlighting questions of interest, corresponding FDA comments, our responses and supporting information, as applicable. We identified a meeting moderator from the core team and technical leads who would actively participate in the meeting. We started our meeting with short introductions of the moderator and core team (5 min) followed by discussion (45 min). The last 10 min were utilized by the NCATS moderator to summarize the meeting and confirm the key takeaways with the FDA. Since sponsor meetings with the Agency cannot be recorded and the FDA does not issue formal INTERACT meeting minutes, it is advisable that sponsors take detailed notes, making certain all outcomes and/or action items reached during the meeting are accurately captured and summarized at the end of the teleconference.

### INTERACT written responses and teleconference major takeaways

**Figure 5.**
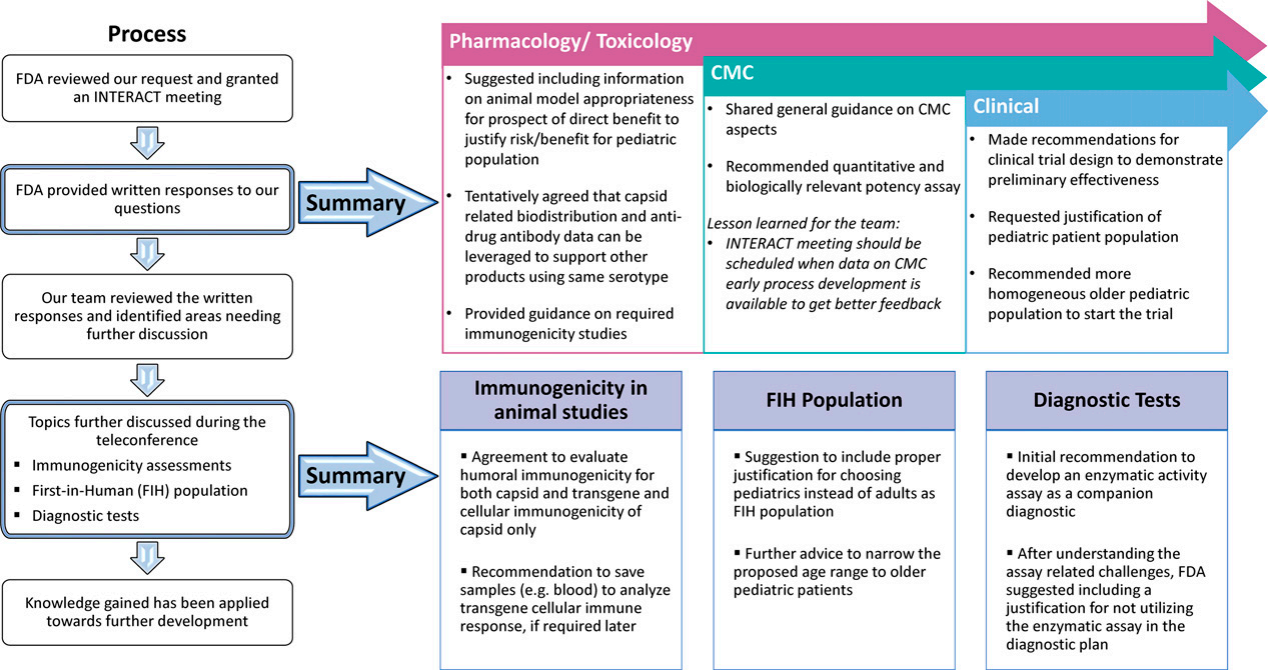
Major takeaways from the AAV9-hPCCA INTERACT meeting experience. The left panel shows the major steps in the FDA process for review of the INTERACT meeting package. Upon reviewing our package, FDA provided feedback in the form of preliminary written responses (top panel). A few selected topics were discussed during the teleconference and major takeaways from the discussion during the INTERACT meeting are listed (bottom panel).

A summary of the preliminary responses prior to the teleconference is provided below and in Figure 5; detailed responses can be found in Supplementary Data S2.

1. FDA gave specific feedback and concurred with our POC data. FDA also gave advice on the type of preclinical immunogenicity information needed for the Pharmacology/toxicology studies. While we presented our proposed, GLP toxicology study plan, FDA deferred review of these studies for later (pre-IND meeting).
2. FDA provided advice on CMC process development, GMP manufacturing, quality attributes, potency assay and device compatibility studies.
3. We received feedback on the clinical trial design, specifically the target population age range.

Our discussions during the INTERACT teleconference revolved around the relevance of transgene immunogenicity testing in the assessment of human safety, disease manifestations and diagnostic tests (Q4 and Q5a, d and e, Supplementary Data S2). We gained further clarity and concurrence from the FDA on these topics (Fig. 5). The feedback gathered from written responses and subsequent teleconference discussion has been helpful to make decisions for our gene therapy program.

Apart from product-specific feedback (Fig. 5), some of the notable learnings related to the PaVe-GT platform approach include:

1. FDA tentatively agreed that capsid-related biodistribution and anti-drug antibody data from the first product can be leveraged to support same serotype products in the PaVe-GT platform within the specified considerations as detailed in File S2 (response to Questions 9).
2. After the teleconference discussion, FDA tentatively agreed that cellular immunogenicity assessment is needed for capsid only, while humoral immunogenicity must be tested for capsid and transgene. In addition, FDA suggested saving blood samples to analyze transgene cellular immune response, if required later.

This feedback is an important step toward incorporating efficiencies as we streamline the development of multiple AAV gene therapies in the PaVe-GT program and for the broader field as well.

## DISCUSSION

The success rate of translating basic science experiments to clinical studies is low due to several challenges^13,17^ and a well-planned drug development program can help to maximize clinical translation. As a sponsor embarks on the journey of developing a new therapeutic, a TPP can serve as a roadmap to guide decision-making and planning for studies at different stages of development. Overall, TPPs can have a positive impact by providing a strategic tool to improve regulatory outcomes.^35–37^ We highly encourage other sponsors to prepare a TPP at the program’s outset, where keeping the end in mind will help them work with regulatory bodies in navigating through preclinical and clinical development.

Given the complexity and unique challenges that each drug development program presents, gaining regulatory concurrence early and consistently in development is beneficial.^38^ Both the TPP and INTERACT meetings helped advance AAV9-hPCCA development, ensuring that preclinical studies are aligned and that regulatory requirements are understood prior to moving the program towards IND-enabling studies. We gained concurrence on our POC data (animal model selection, route of administration and endpoints) in support of the planned FIH studies. Since we had provided limited manufacturing information, FDA shared general CMC advice. While this was instrumental in advancing the manufacturing of our lead candidate, we learned that sharing complete information is essential for getting precise feedback specific to the lead candidate being manufactured. FDA also reviewed our clinical trial design and gave guidance on patient selection, an area of great significance given the struggles with conducting clinical trials in rare diseases affecting the pediatric population. Guidance regarding diagnostic tests and immunogenicity studies has been beneficial in planning the future studies.

**Figure 6.**
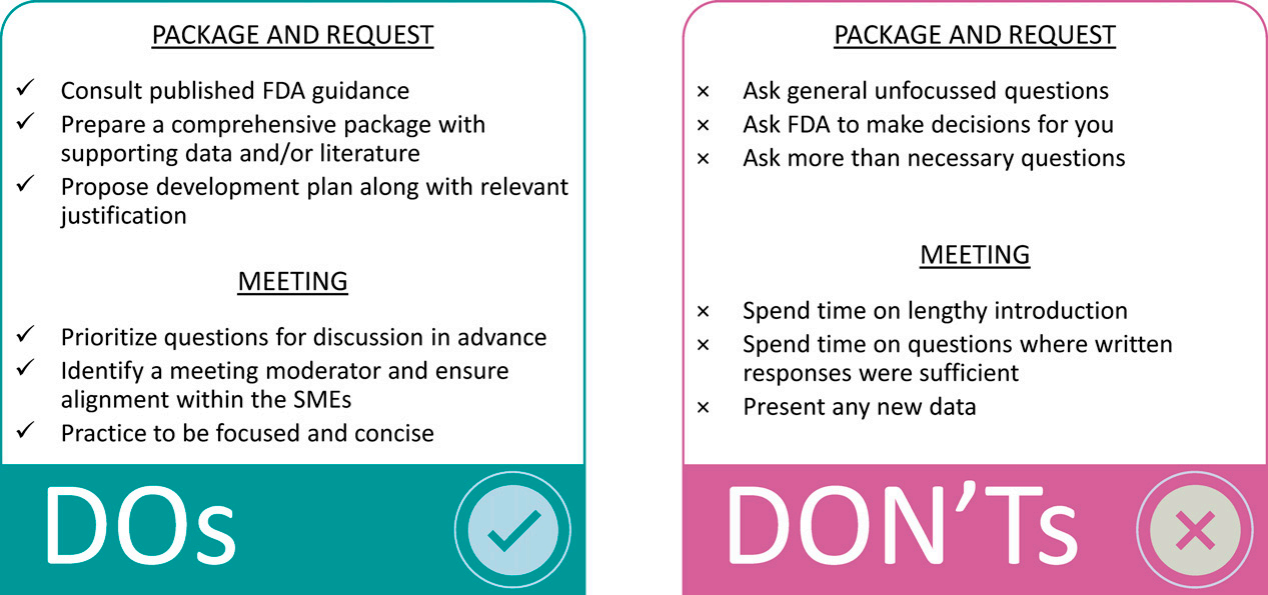
Do’s and don’ts during early regulatory interactions. For a productive discussion, it is important to plan and prepare for regulatory interactions. These are suggested do’s and don’ts based on our experience.

INTERACT meetings were introduced in 2018 and initially limited to CBER;^39^ the recently enacted PDUFA VII extends the INTERACT meetings to CDER as well and categorizes these as formal meetings.^28^ For a productive discussion, it is very important to plan and prepare for regulatory meetings (Fig. 6).^40,41^ Given that only one INTERACT meeting is granted per program, it will be most prudent for the sponsor to focus on product-specific issues and challenges. It is best to refer to available FDA guidance beforehand, formulate questions accordingly, and seek feedback on any issues pertaining to the critical studies. Usually, a thorough package and well-formulated questions will result in precise and valuable FDA feedback. Wherever possible, it is best to formulate questions or present a strategy with rationale in the form of a request and ask for concurrence rather than recommendations or decisions from the FDA.

A key goal of the PaVe-GT program is to test the hypothesis of whether using AAV as a platform vector, and if, by following similar processes, we will be able to incorporate efficiencies and streamline the regulatory path for multiple AAV gene therapies until IND submission. In response to one of our questions, the FDA stated the possibility to leverage capsid-related biodistribution data for the second PaVe-GT product utilizing the same AAV serotype. To benefit the larger AAV community, we also plan to make our regulatory packages including FDA responses publicly available. This illustrates a unique feature of PaVe-GT with our ability to openly share project information because all parties are US government employees. The PaVe-GT program continues to release their experience and learnings to the public. We recently disseminated the regulatory documentation of Orphan Drug and Rare Pediatric Disease Designations, FDA incentives for rare disease drug development.^27^ As the PaVe-GT program moves forward, we will continue to propose and share gene therapy development efficiencies. Experience from subsequent FDA interactions such as pre-IND meeting and IND documentation will be shared as our program advances to those stages in the coming years. Through the sharing of our approach and blueprint of an INTERACT application package, we hope to assist all stakeholders in developing therapeutics for rare diseases.
