# Supplementary material for: Adeno-Associated Virus Gene Therapy Development: Early Planning and Regulatory Considerations to Advance the Platform Vector Gene Therapy Program
Source: Hum Gene Ther. 2025 Mar 6;36(5-6):653–62. doi: 10.1089/hum.2024.230 (PMC11971537; doi:10.1089/hum.2024.230)
Supplement: Supplementary Data S1 [file hum.2024.230_supplementary_datas1.pdf]

AAV9-hPCCA Target Product Profile (TPP)

Note: This TPP for AAV9-hPCCA was developed during early program stages and has since then evolved based on the data obtained and FDA feedback.

Product name: Adeno-Associated Virus 9 vector expressing a functional human codon optimized propionyl carboxylase CoA (PCCA) (AAV9-hPCCA)

Therapeutic Modality: Gene Therapy

**Product Target**	**Minimum Acceptable Result**	**Ideal Result**
**Primary Product Indication and Usage**	Treatment of PCCA-related propionic acidemia (PA) with AAV-driven PCCA transgene and protein expression	Treatment of PCCA-related PA and restoration of clinically meaningful levels of metabolic function, bypassing the need of liver transplantation
**Patient Population**	Pediatric and adolescent patients, aged 2 – 18 years, with PA resulting from a deficiency of PCCA	Pediatric and adult patients, unrestricted in age, with PA resulting from a deficiency of PCCA
**Dosage Form and Stability**	Intravenous infusion; stable for at least 2 years	Intravenous infusion; stable for long term
**Dosing Regimen**	Single-dose intravenous infusion	Single-dose intravenous infusion
**Efficacy/Endpoints**	Stabilization of disease progression<br><br>Change in surrogate endpoints	Improved mortality and morbidity (such as prevention of cardiomyopathy, renal disease, end-organ damage, transplantation, etc) along with subjective measures (patient and caretaker reported outcomes) of quality of life
**Risk/Side Effect**	Devoid of serious adverse events<br><br>Devoid of systemic infusion reaction	Devoid of serious adverse events<br><br>Devoid of systemic infusion reaction
