# Supplementary material for: Adeno-Associated Virus Gene Therapy Development: Early Planning and Regulatory Considerations to Advance the Platform Vector Gene Therapy Program
Source: Hum Gene Ther. 2025 Mar 6;36(5-6):653–62. doi: 10.1089/hum.2024.230 (PMC11971537; doi:10.1089/hum.2024.230)
Supplement: Supplementary Data S2 [file hum.2024.230_supplementary_datas2.pdf]

About the PaVe-GT AAV9-hPCCA INTERACT Meeting Documents

The following documents are communications between the National Institutes of Health (NIH) National Center for Advancing Translational Sciences (NCATS) and the U.S. Food and Drug Administration (FDA) Center for Biologics, Evaluation and Research (CBER) Office of Tissues and Advanced Therapies (OTAT)[1] regarding an INitial Targeted Engagement for Regulatory Advice on CBER Products (INTERACT) meeting. The INTERACT meeting focused on development of AAV9-hPCCA (NCATS-BL0746), a gene therapy for propionic acidemia (PA) resulting from a deficiency of Propionyl-CoA Carboxylase, alpha subunit (PCCA) as part of the Platform Vector Gene Therapy (PaVe-GT) program.

PaVe-GT is a pilot project that will test the hypothesis whether the efficiency of gene therapy trial startup can be significantly improved by using similar processes across gene therapies for four different rare diseases. An important goal of PaVe-GT is to share project results and lessons learned with the public in such a way that the information is useful to any party interested in developing a gene therapy efficiently. Specifically, we will make processes, study results, regulatory documentation and knowledge gained from the PaVe-GT program publicly available. To ensure access to the latest information, please visit the PaVe-GT website, subscribe to project updates, and explore the full set of available resources at pave-gt.ncats.nih.gov/

Some portions of this document—primarily sections that are highly specific to PCCA-related PA and therefore not relevant to other AAV gene therapy efforts—have been edited, redacted or abridged to improve the clarity of materials, and/or support other project objectives. Modified sections are typically identified with italics, brackets, and highlight, [*as shown here*]. The text within the brackets describes the original content. It is important to note that these programs are continually evolving and specifics, such as dosing information, laboratory and animal experiments, analysis, and study duration for all proposed studies may change as results are obtained or feedback is received from the FDA. Some of the data presented in the package below have now been published elsewhere (PMID: 37746248, 31249402, 34007002, 38200289).

Disclaimer: NCATS, NHGRI, and NIH provide no warranties, representations or guarantees that PaVe-GT resources will work for any given project or disease condition. The information is specific to each program, and the following documents are meant to serve only as examples. The mention of trade names, commercial products and organizations does not imply endorsement by the U.S. government. Further, NIH disclaims any liability and provides no indemnification. For a full list of terms and conditions for use of PaVe-GT resources, visit pave-gt.ncats.nih.gov/

[1] After the PaVe-GT AAV9-hPCCA INTERACT meeting, FDA CBER OTAT was reorganized and retitled as the Office of Therapeutic Products (OTP).

Table of Contents for PaVe-GT AAV9-hPCCA INTERACT Meeting Documents

NCATS INTERACT Meeting Request and Package 3

FDA Written Responses 40

NCATS Executive Summary of Teleconference Discussion 64

Attention: Addressed to FDA Review Center/Office Director

Re: Request and briefing package for INTERACT Meeting with CBER/OTAT for Adeno-Associated Virus 9 human Propionyl-Coenzyme A (CoA) Carboxylase, alpha subunit (AAV9-hPCCA), intended for the treatment of pediatric and adult patients, aged 2-18 years, with propionic acidemia (PA) resulting from a deficiency of Propionyl-CoA Carboxylase, alpha subunit (PCCA).

Dear *[Point of contact]:*

Reference is made to the informational meeting held between FDA, Office of Tissues and Advanced Therapies (OTAT), and the National Center for Advancing Translational Sciences (NCATS) on July 2, 2019, regarding the development of the Adeno-Associated Virus 9 (AAV9) Platform Vector-Gene Therapy (PaVe-GT) program established for the treatment of rare diseases using gene therapies (***please see attachment "Supplemental Information")***.

Pursuant to FDA SOPP 8214, titled "*INTERACT Meetings with Sponsors for Drugs and Biological Products,*" NCATS (the Sponsor) is requesting an INTERACT meeting to discuss the investigational gene therapy product, AAV9-hPCCA, intended for the treatment of pediatric and adult patients, aged 2-18 years, with PA resulting from a deficiency of PCCA.

PA is a rare autosomal recessive disorder of organic acid metabolism in humans. It is caused by a deficiency of propionyl-CoA carboxylase (PCC), a ubiquitously expressed, heteropolymeric mitochondrial enzyme involved primarily in the catabolism of propiogenic amino acids, particularly isoleucine, valine, methionine, and threonine, as well as odd-chain fatty acids. The enzyme is composed of α- and β-subunits encoded by their respective genes, *PCCA* and *PCCB*.

Most frequently, PA presents in the neonatal period with hyperammonemia, vomiting, poor feeding and hypotonia, and progresses into a life-threatening metabolic crisis. Patients who survive suffer from recurrent metabolic instability and can develop multisystem complications, including cardiomyopathy. The long-term prognosis for survival in severely affected patients is poor, where PA patients with an early and severe clinical course experience increased mortality and disease associated morbidity. The recalcitrant nature of the disorder to conventional medical management, including the dietary restriction of amino acid precursors, L-carnitine supplementation, and administration of metronidazole to reduce the generation of propionic acid by intestinal bacteria, has led to the implementation of elective liver transplantation (LT) as an experimental surgical treatment for PA. While not curative of all aspects of the disorder, successful LT in the setting of PA provides restoration of metabolic stability and protection from early death, and therefore represents a clinical benchmark for gene replacement therapy that may increase hepatic PCC expression and activity. There is currently no approved FDA drug or biologic for the treatment of PA.

Included in this meeting package, please find our INTERACT meeting request for a teleconference, along with the briefing package describing a synopsis of the proposed clinical study, summary of the development and preclinical work, proposed IND-enabling studies, a brief description of the manufacturing process for the investigational drug product, and specific questions we would like the Agency to address.

NCATS is requesting the following CBER OTAT representatives be present at the meeting:

1. Pharmacotoxicology reviewer
2. CMC reviewer
3. Clinical reviewer

I, *[Point of contact],* will serve as the primary contact on this submission. Furthermore, I authorize the following individuals to receive FDA communications regarding this submission: *[Name and contact information of three individuals was included]*

For any questions regarding this submission, please contact me at *[contact information],* including my listed authorized contacts above.

Sincerely,

[*Signature/ contact information*]

TABLE OF CONTENTS

[The page numbers below have been adjusted]

1. MEETING REQUEST/PACKAGE ... *9*

1.1. Product Name ... **9**

1.2. Application Number ... **9**

1.3. Chemical Name and Structure ... **9**

1.4. Proposed Indication ... **9**

1.5. Type of Meeting ... **9**

1.6. Brief Statement of the Purpose and Objectives of the Meeting ... **9**

1.7. The Format of the Meeting ... **9**

1.8. Suggested Dates and Times ... **10**

1.9. Proposed Agenda ... **10**

1.10. List of All Individuals Who Will Attend the Proposed Meeting ... **10**

1.11. List of Questions ... **11**

2. A SUMMARY OF THE DATA TO SUPPORT DISCUSSION ... *15*

2.1. Nonclinical ... **15**

2.1.1. AAV9-hPCCA as a Potential Treatment for PA ... 15

2.1.2. Published PA Mouse Models and Gene Therapy Studies ... 16

2.1.3. Studies in a Lethal Mouse Model of PA Used in the Current Program ... 17

2.1.4. Proposed GLP Toxicology Study ... 22

2.2. Chemistry, Manufacturing and Controls ... **24**

2.3. Clinical ... **25**

2.3.1. Proposed Phase 1/2 Clinical Trial Protocol Synopsis ... 30

2.4. PaVe-GT Platform Program ... **34**

2.5. Planning for Expedited Pathways ... **35**

3. REFERENCES ... *37*

APPENDIX A ... *38*

APPENDIX B ... *39*

LIST OF TABLES

Table 1. Survival and Metabolic Phenotype Summary of *Pcca*$^{-/-}$ Animals Treated with Different Doses of AAV9-hPCCA ... 17

Table 2. *Pcca*$^{-/-}$ Neonatal Knockout Mouse Model ... 17

Table 3. List of AAV9-hPCCA Treated Neonatal *Pcca*$^{-/-}$ Mice. ... 18

Table 4. GLP 6-Month Toxicity and Biodistribution Study Design ... 23

LIST OF FIGURES

Figure 1. Catabolism of Propionyl-CoA.......... 15
Figure 2. Survival Curve of AAV9-hPCCA Treated *Pcca*$^{-/-}$ Mice.......... 19
Figure 3. Plasma 2-MC Levels in AAV9-hPCCA Treated *Pcca*$^{-/-}$ Mice. 20
Figure 4. Hepatic *PCCA* mRNA and PCCA Protein Expression in AAV9-hPCCA Treated *Pcca*$^{-/-}$ Mice 21
Figure 5. Biodistribution of AAV9-hPCCA in Liver and Heart Tissues 22
Figure 6. A Schematic of the Vector Transgene and Description of the Cassette. 24
Figure 7. Clinical and Laboratory Manifestations of PA 25
Figure 8. Clinical Characteristics of the NIH Natural History Cohort. 26
Figure 9. Age-dependent Manifestations of PA Supporting Pediatric Use of Biologics Aiming to Restore Propionate Oxidation.......... 27
Figure 10. Performance of PA Diagnostic Variables as Response Biomarkers Depends on the Renal Status and Levocarnitine Supplementation. 28
Figure 11. *In Vivo* 1-^{13}C-Propionate Oxidation Correlates with Diagnostic Biomarkers, Clinically Relevant Outcomes and Liver Transplant Status, and Does Not Depend on the Renal Function.... 29

LIST OF ABBREVIATIONS

AAV	Adeno-Associated Virus
AAV2	Adeno-Associated Virus serotype 2
AAV8	Adeno-Associated Virus serotype 8
AAV9	Adeno-Associated Virus serotype 9
AAV9-hPCCA	Adeno-Associated Virus 9 human Propionyl-CoA Carboxylase, alpha subunit
ADA	Anti-Drug Antibody
BGH	Bovine Growth Hormone
Cas9	CRISPR associated protein 9
CMC	Chemistry, manufacturing, and controls
CMO	Contract Manufacturing Organization
CoA	Coenzyme A
ColQ	Collagen Q
CRISPR	Clustered regularly interspaced short palindromic repeats
CRM	Cross Reactive Material
CRO	Contract Research Organization
DOK7	Downstream of Tyrosine Kinase 7
DSMB	Data and Safety Monitoring Board
ELISpot	Enzyme-Linked Immunosorbent Spot
F	Female

FIH	First in human
FT	Fast Track
GLP	Good Laboratory Practice
GMP	Good Manufacturing Practice
HPRE	Hepatitis B posttranslational regulatory element
IND	Investigational New Drug
ITR	Inverted Terminal Repeat
IV	Intravenous
LT	Liver Transplantation
M	Male
2-MC	2-Methylcitrate
MCEE	D-methylmalonyl-CoA epimerase
MMAB	Methylmalonic acidemia type B
MMUT	Methylmalonyl-CoA mutase
mRNA	Messenger Ribonucleic Acid
NCATS	National Center for Advancing Translational Sciences
NHGRI	National Human Genome Research Institute
NIH	National Institutes of Health
NINDS	National Institute of Neurological Disorders and Stroke
NINR	National Institute of Nursing Research
OAA	Oxaloacetic acid
P	Postnatal Day
PA	Propionic Acidemia
PAVE-GT	Platform Vector-Gene Therapy
PBS	Phosphate Buffered Saline
PCC	Propionyl-CoA Carboxylase (EC 6.4.1.3)
Pcca	Propionyl-CoA Carboxylase, alpha subunit gene (mouse)
PCCA	Propionyl-CoA Carboxylase, alpha subunit protein
PCCA	Propionyl-CoA Carboxylase, alpha subunit gene (human)
PCCB	Propionyl-CoA Carboxylase, beta subunit gene (human)
POC	Proof of Concept
qPCR	Quantitative Polymerase Chain Reaction
RMAT	Regenerative Medicine Advanced Therapy
RNA	Ribonucleic acid

ROA	Route of Administration
RT-PCR	Reverse Transcription Polymerase Chain Reaction
US	United States
vg	vector genome
WT	Wild Type

1. MEETING REQUEST/PACKAGE

1.1. Product Name

Adeno-Associated Virus 9 human Propionyl-CoA Carboxylase, alpha subunit (AAV9-hPCCA).

1.2. Application Number

To be assigned by Center for Biologics Evaluation and Research (CBER), Office of Tissues and Advanced Therapies (OTAT).

1.3. Chemical Name and Structure

AAV9-hPCCA is an Adeno-Associated Virus 9 vector expressing a functional human codon optimized cDNA encoding the Propionyl-CoA Carboxylase, alpha subunit (*PCCA*), under control of *[a specific promoter]*. Details describing the vector transgene are found in ***Section 2.2***.

[Schematic describing components of the AAV9-hPCCA cassette]

1.4. Proposed Indication

Treatment of pediatric and adult patients aged 2-18 years, with propionic acidemia (PA) resulting from a deficiency of Propionyl-CoA Carboxylase, alpha subunit (PCCA).

1.5. Type of Meeting

Initial Targeted Engagement for Regulatory Advice on CBER Products (INTERACT).

1.6. Brief Statement of the Purpose and Objectives of the Meeting

The purpose of this meeting is to gain feedback and concurrence from the Agency on the proposed preclinical proof-of-concept (POC), pharmacology/toxicology, and clinical and regulatory aspects of the AAV9-hPCCA development program for the initiation of the first in human (FIH) study. We have also included high level chemistry, manufacturing, and controls (CMC) information, where any general feedback from the Agency would be appreciated. In addition, we would like to gain feedback and concurrence from the Agency on the NCATS Platform Vector Gene Therapy (PaVe-GT) program, whereby the presented AAV9-hPCCA development project is the first to utilize this platform-based approach.

1.7. The Format of the Meeting

One-hour teleconference.

1.8. Suggested Dates and Times

Due to the novelty of our approach, we are requesting a teleconference meeting with FDA review team and management on either Tuesday, May 4, 2021, Thursday, May 6, 2021, or Friday, May 7, 2021.

1.9. Proposed Agenda

**Topic**	**Estimated Duration**
Introductions	5 minutes
Discuss Agency responses to specific Regulatory, Nonclinical, Clinical, and PaVe-GT platform questions	50 minutes
Summarize key agreements	5 minutes

1.10. List of All Individuals Who Will Attend the Proposed Meeting

[List of participants with title and affiliation]

1.11. List of Questions

Nonclinical

For testing of AAV gene therapy vectors for human translation, we generated mice using CRISPR/Cas9 genome editing to engineer mutations that are compatible with those seen in the patients, such as frameshift-stop and missense changes. The mutation, Pcca $^{p.Q133LfsX41}$, caused by a 4 bp deletion in the *Pcca* gene, is severe and null at the level of Pcca protein expression (cross reactive material, CRM-). Homozygous mice (*Pcca*$^{-/-}$) with this mutation were generated and recapitulate the neonatal lethal form of PA in humans. These mice perish in the immediate neonatal period (within 24 hours of birth) without gene therapy treatment, and hence, are particularly useful to assay the efficacy of AAV9-hPCCA gene therapy for the treatment of PA. Data from the *in vivo* studies demonstrate that administration of AAV9-hPCCA vector rescues *Pcca* $^{-/-}$ neonatal mice (***Section 2.1.3***). We are proposing to establish the first-in-human (FIH) dosing based on the data obtained from the *Pcca*$^{-/-}$ neonatal mouse model studies.

Regarding the planned Good Laboratory Practice (GLP) toxicology and biodistribution study (outsourced to the Contract Research Organization (CRO), Lovelace Biomedical), we intend to use a Good Manufacturing Practice (GMP) manufactured batch of AAV9-hPCCA. Briefly, we intend to dose 8 week old wild type (WT) mice, with AAV9-hPCCA at a dose of up to 1 x 10^{14} vg/kg via tail vein injection, to support the proposed peripheral intravenous or peripherally inserted central catheter (PICC) line clinical route of administration (ROA) in the human study. Please see details of the proposed toxicology study under ***Section 2.1.4***. No non-rodent toxicology studies are planned for toxicology.

1. **Does the Agency agree that the efficacy observed in the proof of concept (POC) studies in the *Pcca*$^{-/-}$ neonatal mouse model (*Section 2.1.3*) is sufficient to support proposed clinical testing?**
2. **Does the agency agree with our proposal of establishing the FIH dosing based on the *Pcca*$^{-/-}$ neonatal mouse model studies (*Section 2.1.3*)?**
3. **Does the Agency agree with the proposed toxicology study design in WT mice, specifically:**
    a. **Does the Agency agree with the proposed toxicology dose of up to 1 x 10^{14} vg/kg in support of the proposed FIH doses?**
    b. **Does the Agency agree that the GLP toxicology study ROA, tail vain injection in 8 week WT mice, is sufficient to support our proposed FIH human trial using either the IV or PICC line ROA?**
4. **For the immunogenicity assessment portion of the planned toxicology study, we plan to only perform *in vivo* anti-AAV9 capsid antibody testing. We will not conduct any additional immunogenicity assessments, such as ELISpot assays in WT animals or mouse antibodies against PCCA, since the mouse immune responses to the encoded transgene may not be informative towards human translation. Does the Agency agree?**

Chemistry, Manufacturing and Controls (CMC)

AAV9-hPCCA will be manufactured using standard production methods for AAV9 vectors. To date, our contract manufacturing organization (CMO), Vigene Biosciences, Inc. (Vigene), has manufactured a small-scale feasibility batch (final report pending), and we project that Vigene will also manufacture the GMP grade investigational drug product. Briefly, Vigene has demonstrated experience in the GMP manufacturing of investigational gene therapy products for clinical trials. Details about the transgene and vector can be found in ***Section 2.2***. We will provide the Agency all supporting manufacturing information in our planned pre-IND briefing package. Any general CMC related guidance from the Agency would be appreciated.

Clinical/Regulatory

PA is a life-threatening autosomal recessive metabolic disorder caused by deleterious variants in either the *PCCA* or *PCCB* genes, which lead to the impaired activity of ubiquitously expressed PCC. The clinical course of PA is characterized by chronic multi-organ dysfunction punctuated by episodes of metabolic instability due to metabolic acidosis, ketonuria, hyperammonemia, and hypoglycemia precipitating emergency rooms visits and hospitalizations (Shchelochkov et al., 2012 [updated *2024*]). Standard of care includes life-long dietary protein restriction, supplementation with L-carnitine and supportive treatment. Currently, there are no FDA approved drugs or biologics available to offer patients with this life-threatening disease.

Because of the poor prognosis for long term survival, metabolic instability, and progression of disease experienced by patients as they age, elective liver transplantation (LT) has been used as a surgical treatment for PA patients. Patients who underwent LT experience fewer metabolic ketoacidotic episodes and show improved dietary protein tolerance. LT has been associated with improved blood biomarkers reflecting improved metabolic control of PA and therefore, represents a clinical benchmark for gene replacement or additional approaches that might increase hepatic PCC expression and activity. Nevertheless, organ availability, surgical complications, and toxic side effects of life-long immunosuppression after LT remain as practical constraints to the widespread adoption of LT as a preferred treatment for PA.

We are proposing an open-label Phase 1/2 dose-escalation study (three dose cohorts) of systemic AAV9-hPCCA gene therapy in up to 10 pediatric and adult PCCA patients, aged 2-18 years (***Section 2.3.1***). Our proposed FIH starting dose of *[low dose]* vg/kg is based on the efficacy observed in the POC $Pcca^{-/-}$ neonatal mice studies, where partial rescue from lethality was observed at 8.3 x 10^{12} vg/kg (***Figure 2***). The proposed FIH starting dose of *[low dose]* vg/kg is further supported by the clinical dose ranges of 2 x 10^{13} vg/kg to 1 x 10^{14} vg/kg used in the MPSIIIB clinical trial, another systemic gene therapy using AAV9 vector in a human trial of inborn errors of metabolism (***NCT03315182***). The primary endpoint is safety and tolerability, with secondary endpoints as changes from baseline in the response biomarkers *[two disease related biomarkers]* concentrations. The response biomarkers will be collected

per protocol synopsis (*Section 2.3.1*) and will be used to make dose escalation decisions (in addition to the safety assessments). Patients will undergo an additional 5-years of long-term follow up for safety and laboratory monitoring.

5. **Regarding the proposed clinical synopsis (*Section 2.3.1*), does the Agency concur with the following:**
   a. **The proposed study design?**
   b. **The safety and sufficiency of a proposed initial human dose of *[low dose]* vg/kg for FIH testing?**
   c. **The proposed three dose cohorts and dose escalation plan?**
   d. **The FIH population with an age range of participants from 2 to 18 years?**
   e. **The oral prednisone (1 mg/kg/day) for 4-6 weeks is appropriate to mitigate possible immune response to administration of AAV9-hPCCA?**
   f. **The primary and secondary endpoints in the proposed clinical study?**
   g. **The study duration and the proposed intervals for biomarker and preliminary efficacy testing?**
6. Subacute complement activation following systemic AAV9 administration in boys with DMD was reported in the Pfizer (***NCT03362502***), 2 dose levels: $1x10^{14}$ vg/kg n=3 and 3e14 vg/kg n=6. Weight range 18-42 kg) and Solid (***NCT03368742***), 2 dose levels $5x10^{13}$ vg/kg and $2x10^{14}$ vg/kg. 3 patients per dose cohort) trials resulting in clinical holds. Patients presented with rapid thrombocytopenia with evidence for complement activation as evidenced by C3 and C4 complement consumption and rise in C5b-9 within 24 hours as well as evidence for microangiopathy. In the Pfizer trial one patient suffered from acute renal injury requiring dialysis and was treated with eculizumab, and another participant received platelet transfusions and eculizumab. Both had no detectable pre-existing AAV9 neutralizing antibodies but in at least one of them there was a rapid and dramatic rise in AAV9 neutralizing antibody titer within the first week post dosing suggesting formation of antibody/capsid complexes at higher total AAV loads as a trigger for complement mitigation. Similar events have occurred in the Solid trial, again prompting the use of eculizumab (*NIH NCATS/FDA CBER Workshop on Systemic Immunogenicity Considerations for AAV-Mediated Gene Therapy, November 30 - December 1, 2020*).

   In light of recent safety signals related to the subacute complement cascade activation in recent trials of systemic high dose AAV9 mediated gene transfer in boys with DMD, does the Agency have any concerns and/or feedback regarding the proposed clinical protocol synopsis, risk mitigation strategy and AAV9-hPCCA development plan in the planned FIH study?
7. **Based on the presented POC animal studies and supporting rationale, outlined under *Section 2.5*, Planning for Expedited Pathways, does the Agency agree with our proposal to submit a Fast Track (FT) designation request at time of IND submission?**
8. **We are proposing submission of a Regenerative Medicine Advanced Therapy**

Designation (RMAT) designation request within the first two months of the Phase 1/2 FIH study utilizing response biomarker data (*Section 2.5*). Does the Agency agree with the proposed timing of RMAT designation request?

PaVe-GT Platform

Platform Vector Gene Therapy (PaVe-GT) is a program whose main goal is to test whether the efficiency of gene therapy development and clinical testing can be substantially increased by using a standardized process for four different rare diseases (Brooks et al., 2020). PaVe-GT will use the same AAV serotype, AAV-9, as a platform vector to develop gene therapy products for the four selected rare diseases. The rare monogenic diseases selected include two organic acidemias (liver) and two congenital myasthenic syndromes (neuromuscular junction). The organic acidemias under study are PA (caused by PCCA deficiency) and isolated methylmalonic acidemia (MMAB deficiency/cobalamin type B methylmalonic acidemia). The congenital myasthenic syndromes under study are characterized by deficiency of the protein DOK7 (downstream of tyrosine kinase 7) and deficiency of Collagen Q (ColQ), a specific nonfibrillar collagen.

AAV9 was selected for this project because of its broad cell and tissue tropism, including the liver, heart, muscle, and the central nervous system. The AAV9 vector used in clinical trials here will be produced in the same manufacturing facility, using the same production and purification methods, with the only difference being the therapeutic gene constructs. While POC studies will be conducted individually for the four selected rare disorders, we hypothesize that in addition to CMC streamlining, efficiencies will be gained in biodistribution, and potentially toxicology studies, as the preclinical testing advances.

9. **As per our understanding from our meeting with the Agency on July 2, 2019 and FDA Guidance for Industry, "*Preclinical Assessment of Investigational Cellular and Gene Therapy Products*", November 2013, is the Agency still in agreement that the planned biodistribution studies for AAV9-hPCCA would be sufficient to support preclinical development programs for subsequent gene targets, utilizing our established AAV9 delivery capsid platform (i.e., different gene target and intended population)? We acknowledge that the Agency would like for us to submit a separate IND for each of the four rare disease indications discussed above.**
10. **Does the Agency agree that the planned preclinical anti-drug antibody (ADA) measured against the AAV9 capsid in the AAV9-hPCCA toxicology study, would be sufficient to support subsequent planned gene therapy development programs utilizing the same AAV9 capsid under a different IND (i.e., different gene target and intended population)?**
11. **Can the Agency provide feedback on whether a drug master file/s (DMF) would be recommended for the PaVe-GT project?**

2. A SUMMARY OF THE DATA TO SUPPORT DISCUSSION

2.1. Nonclinical

2.1.1. AAV9-hPCCA as a Potential Treatment for PA

PA is a well-recognized autosomal recessive disorder of organic acid metabolism in humans with an estimated incidence of 1:250,000-1:750,000 births. It is caused by a deficiency of PCC, a ubiquitously expressed, heteropolymeric mitochondrial enzyme involved primarily in the catabolism of propiogenic amino acids, particularly isoleucine, valine, methionine, and threonine, as well as odd-chain fatty acids. The enzyme is composed of α- and β-subunits encoded by their respective genes, *PCCA* and *PCCB*. PCC catalyzes the first step in the conversion of propionyl-CoA to D-methylmalonyl-CoA in the pathway of propionyl-CoA oxidation, depicted in ***Figure 1***. PA is caused by mutations in either the *PCCA* or *PCCB* gene. The formation of 2-MC, an important biomarker generated through the condensation of oxaloacetic acid (OAA) and propionyl-CoA is also noted, as are downstream enzymatic steps in the pathway, including D-methylmalonyl-CoA epimerase (MCEE) and methylmalonyl-CoA mutase (MMUT), in the metabolism of propionyl-CoA into the citric acid (Krebs) cycle.

Figure 1. Catabolism of Propionyl-CoA.

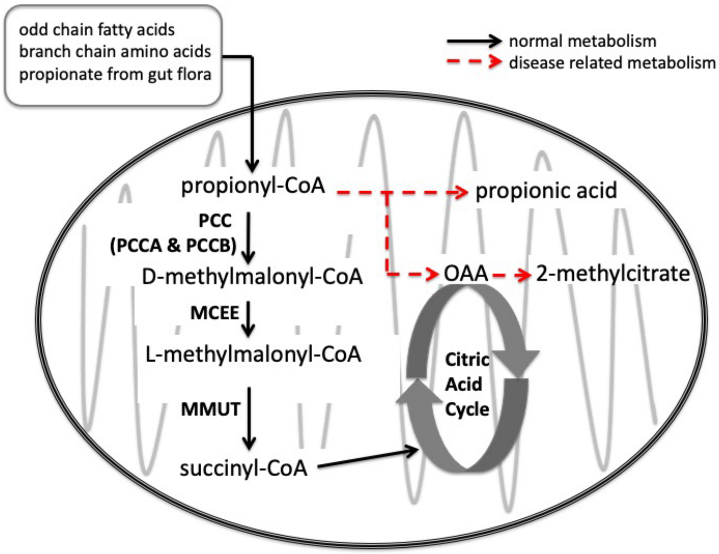

Most frequently, PA presents in the neonatal period with hyperammonemia, vomiting, poor feeding, and hypotonia and progresses into a life-threatening metabolic crisis. Patients who survive suffer from recurrent metabolic instability and can develop multisystemic complications, including cardiomyopathy. The long term prognosis for survival in severely affected patients is poor as illustrated by an early and relatively large (for the disease prevalence) single center study of 20 patients with PA treated at a tertiary care center: Those who presented in the first week of life (11

patients) largely perished by the age of 6 years (Surtees et al., 1992). Over the decades, it has been recurrently noted that PA patients with an early and severe clinical course experience increased mortality and disease associated morbidity (Shchelochkov et al., 2012 [updated 2016, *[now updated 2024*]). The recalcitrant nature of the disorder to conventional medical management, including the dietary restriction of amino acid precursors, L-carnitine supplementation, and administration of metronidazole to reduce the generation of propionic acid by intestinal bacteria, has led to the implementation of elective LT as an experimental surgical treatment for PA. While not curative of all aspects of disorder, successful LT in the setting of PA provides restoration of metabolic stability and protection from early death, and therefore represents a clinical benchmark for gene replacement or addition approaches that might increase hepatic PCC expression and activity.

2.1.2. Published PA Mouse Models and Gene Therapy Studies

The first murine model reported was generated by targeted disruption of the *Pcca* gene via homologous recombination in embryonic stem cells (Miyazaki et al., 2001). This allele, which deletes several kilobase pairs within the *Pcca* gene, behaves as a null mutation, with mice homozygous for the deletion in the *Pcca* gene ($Pcca^{del/del}$) perishing within the first 48 hours of birth. The earliest gene therapy studies in this model of PA yielded disappointing results. Treatment with an adenoviral vector designed to express the human PCCA cDNA showed only a fractional increase in the life expectancy (Hofherr et al., 2009). Hofherr, et.al. also reported that AAV serotype 8 (AAV8)-mediated gene transfer of PCCA was ineffective, which was unexpected. However, a later study, using the same mouse model and informed by a series of successful studies with mouse models of methylmalonic acidemia, demonstrated that systemic delivery of an AAV8 vector that expressed the human *PCCA* gene under the control of a strong heterologous promoter provided effective rescue from neonatal lethality, restoration of hepatic PCC expression, and substantial reduction in the level of the PA associated biomarker, 2-MC, in the blood of the $Pcca^{del/del}$ mice (Chandler et al., 2011). Furthermore, a pilot study in the AAV8 treated $Pcca^{del/del}$ mice demonstrated intermediate oxidization of 1-^{13}C propionic acid into $^{13}CO_2$ after the gene therapy compared to wild type (WT) control littermates, suggesting a monitoring approach for AAV-mediated hepatic correction of PCCA that could one day be extended to human subjects (Chandler et al., 2011).

The fragility of the $Pcca^{del/del}$ mice led to the development of a mildly affected or hypomorphic animal model of PA (Guenzel et al., 2013). These investigators created transgenic mice that overexpress a human mutation $PCCA^{p.A138T}$ (the transgene is designated $Tg^{PCCA\ p.A138T}$) associated with ~10% of PCC enzyme activity and bred the transgenic mice to create $Pcca^{del/del}$; $Tg^{PCCA\ p.A138T}$, a hypomorphic mouse model of PA. The resulting mice are largely rescued from PA by the transgenic overexpression of $PCCA^{p.A138T}$, and are very mildly affected, displaying intact growth, preserved survival, and only modest elevations of biochemical markers of the disease. Even though this model does not recapitulate the most salient clinical feature of PA (lethality), there are published reports using $Pcca^{del/del}$; $Tg^{PCCA\ p.A138T}$ mice to test adenoviral and AAV PCCA vectors (serotypes AAV8, AAV2, and AAVrh10) for therapeutic efficacy by assaying the reduction in the

levels of metabolites after gene delivery (Guenzel et al., 2015; Guenzel et al., 2014; Guenzel et al., 2013). The gaps between clinically relevant Pcca alleles and mutations seen in patients, and uncertain efficacy of AAV vectors to treat severe PA led us to create new Pcca mouse models and design improved vectors for human translation.

2.1.3. Studies in a Lethal Mouse Model of PA Used in the Current Program

We have conducted a series of studies in a newly generated knockout mouse model, designated *Pcca*$^{-/-}$ and detailed below. The mice have no detectable Pcca protein and display immediate neonatal lethality, akin to the phenotype of the human disorder [*Chandler, R.J., et.al. 2023*]. A summary of the POC studies conducted in *Pcca* $^{-/-}$ neonatal mice after administration of AAV9-hPCCA thus far is presented below in ***Table 1***. The gene therapy shows a pronounced effect on the animal survival and reduction in the plasma levels of the pathological metabolite, 2-MC.

Table 1. Survival and Metabolic Phenotype Summary of *Pcca*$^{-/-}$ Animals Treated with Different Doses of AAV9-hPCCA

Treatment Group	Dose (vg/kg)	No. of *Pcca*$^{-/-}$ Mice	Mean Survival (Days) ± Standard Deviation	Metabolic Phenotype (2-MC)
Untreated	N/A	24	1.6±0.8	+++ (> 50 mM)
PBS	N/A	11	1.0±0.0	N.D.
*AAV9-hPCCA	8.3 x 10^{12}	4	41.0 ±26.7	++ (> 20 mM)
*AAV9-hPCCA	8.3 x 10^{13}	31	55.9 ±42.5	+ (< 20 mM)
*AAV9-hPCCA	3.3 x 10^{14}	12	112.5±52.5	+ (< 20 mM)

<u>*Note:</u> Different lots of research grade AAV9-hPCCA were used for the *Pcca* $^{-/-}$ studies listed in the table above, but all contained the same therapeutic transgene, and all animals were dosed by retro-orbital injection on post-natal day 1 (P1).

Based on the accumulative POC results from the *Pcca* $^{-/-}$ neonatal knock out mouse model, we are proposing to test up to 1x10^{14} vg/kg in the planned GLP toxicology study.

Experimental Design and Results

For definitive testing of AAV gene therapy vectors for human translation, we created mice using CRISPR/Cas9 genome editing to engineer mutations that are compatible with those seen in the patients, such as frameshift-stop and missense changes. One mutation, Pcca$^{p.Q133LfsX41}$ in Exon 5 of the *Pcca* gene is severe and null at the level of protein expression (CRM -). Please see ***Table 2***.

Table 2. *Pcca*$^{-/-}$ Neonatal Knockout Mouse Model

Pcca exon Targeted	Pcca Protein Mutation	Homozygous phenotype
5	p.Q133LfsX41	Neonatal lethal<br>CRM negative<br>Increased 2-MC

Building on many years of previous work with AAV gene therapy in related mouse models of methylmalonic acidemia, where early lethality is a uniform characteristic of the homozygous mutant phenotype, we initiated our studies to determine the therapeutic efficacy of AAV9-hPCCA delivered by retro-orbital plexus injection in newborn *Pcca*$^{-/-}$ mice. Tail vein injections are not possible in neonatal mice on P1, and facial vein injections are most easily accomplished on P2. Because the untreated *Pcca*$^{-/-}$ mice experience 100% lethality by P2 (***Figure 2***), we injected AAV9-hPCCA via retro-orbital plexus to the systemic circulation, to recapitulate IV or PICC line delivery, the anticipated ROA in humans.

Survival of AAV9-hPCCA Treated *Pcca*$^{-/-}$ Mice

Homozygous *Pcca*$^{-/-}$ males rescued by AAV9 gene delivery were mated with heterozygous animals. All the pups obtained in the litter were treated with AAV9-hPCCA within a few hours after birth (at P0-1). For the survival study in the *Pcca*$^{-/-}$ mice, we tested three doses of AAV9-hPCCA, 8.3 x10^{12} vg/kg (n=4), 8.3 x10^{13} vg/kg (n=31) and 3.3 x10^{14} vg/kg (n=12), administered via retro-orbital injections. To minimize stress on the newborns and mothers, the pups were not weighed before AAV9-hPCCA administration, the vector was dosed as vg/pup and approximated for vg/kg using the average body weight of 1.2g (n=20). The genotypes of all pups were not determined until after injection. A complete list of all dosed pups (including the different genotypes, approximately 200 animals) is presented in ***Appendix A***.

Table 3 summarizes the doses administered to the homozygous *Pcca*$^{-/-}$ neonatal animals. Subsequently, survival was monitored, and the transgene expression was measured. At selected timepoints, biomarker responses (2-MC) and vector genome biodistribution were measured.

Table 3. List of AAV9-hPCCA Treated Neonatal *Pcca*$^{-/-}$ Mice.

Genotype	Treatment	Dose (vg/pup)	Dose (vg/kg)	Sex	Number of Mice
Pcca$^{-/-}$	None	N/A	N/A	Not Available	24
Pcca$^{-/-}$	PBS	N/A	N/A	Not Available	11
Pcca$^{-/-}$	AAV9-hPCCA	1 x10^{10}	8.3 x10^{12}	M	1
Pcca$^{-/-}$	AAV9-hPCCA	1 x10^{10}	8.3 x10^{12}	F	2
Pcca$^{-/-}$	AAV9-hPCCA	1 x10^{10}	8.3 x10^{12}	Not Available	1
Pcca$^{-/-}$	AAV9-hPCCA	1 x10^{11}	8.3 x10^{13}	M	11
Pcca$^{-/-}$	AAV9-hPCCA	1 x10^{11}	8.3 x10^{13}	F	13
Pcca$^{-/-}$	AAV9-hPCCA	1 x10^{11}	8.3 x10^{13}	Not Available	7
Pcca$^{-/-}$	AAV9-hPCCA	4 x10^{11}	3.3 x10^{14}	M	4
Pcca$^{-/-}$	AAV9-hPCCA	4 x10^{11}	3.3 x10^{14}	F	8

The results for the various dose cohorts of AAV9-hPCCA treated *Pcca* $^{-/-}$ mice are presented in ***Figure 2*** below. All three doses provide rescue from neonatal lethality, and substantially prolong the life of the mutant mice in a dose response manner. The control *Pcca* $^{-/-}$ animals (untreated/ PBS treated) died shortly after birth. The animals dosed at 8.3 x10^{12} vg/ kg had a mean survival of ~ 41 days, which increased with dose: those in the 8.3 x10^{13} vg/ kg cohorts survived for ~ 55.9 days, while animals in the highest dose group of 3.3 x10^{14} vg/ kg lived even longer, with a mean survival past 112.5 days, with some animals still alive at the time of submission of the meeting request, clearly demonstrating the efficacy of AAV9-hPCCA gene therapy treatment. In addition, 53 *Pcca* $^{+/-}$ (heterozygous) mice were treated with the same doses as the *Pcca* $^{-/-}$ (homozygous) mice (***Appendix A***). The AAV9-hPCCA treated *Pcca* $^{+/-}$ mice were followed until day 30 and appear healthy in comparison to untreated *Pcca* $^{+/-}$ mice.

Figure 2. Survival Curve of AAV9-hPCCA Treated *Pcca*$^{-/-}$ Mice.

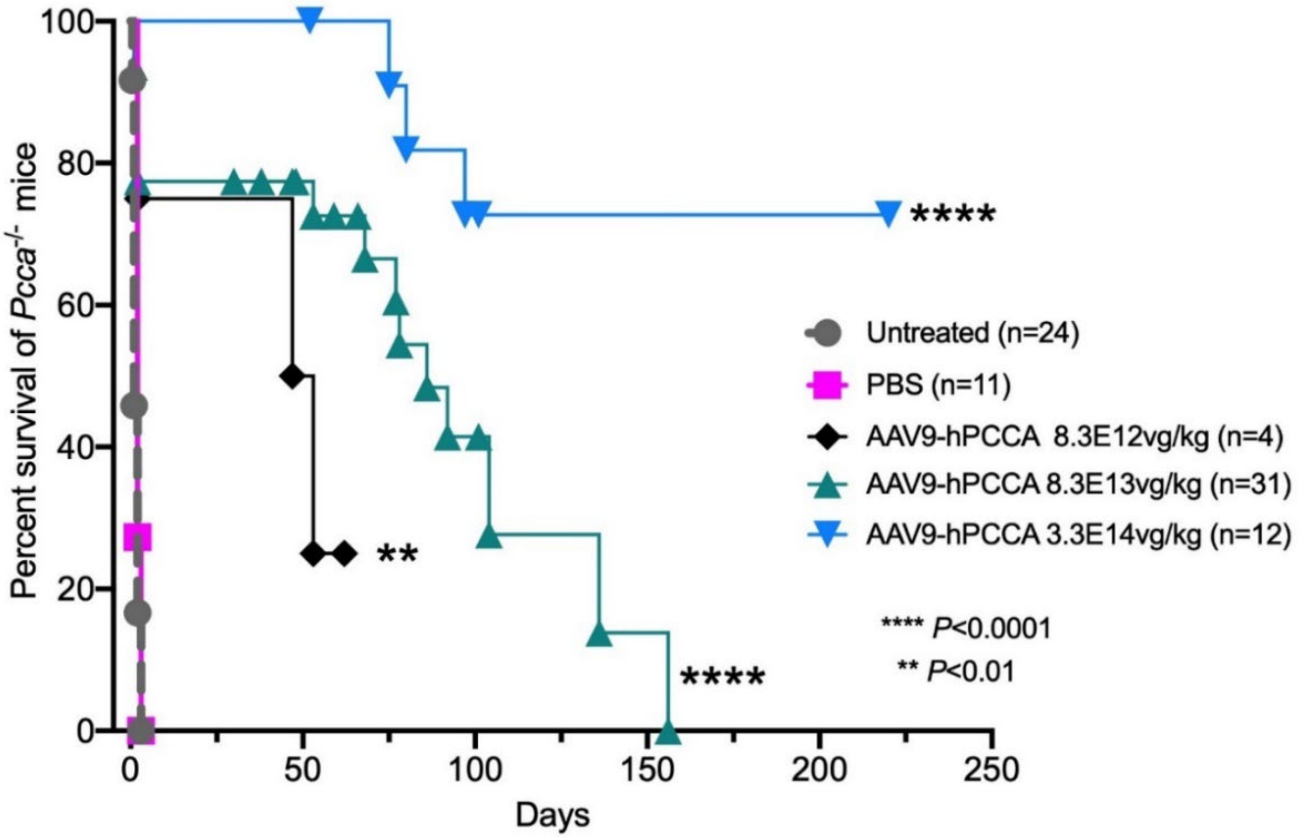

Mice were treated with indicated doses of AAV9-hPCCA at P0-1. PBS and no treatment (untreated) were used as a control. The graph depicts the percent survival of different cohorts of animals ** *P*<0.0001, ** *P*<0.01 compared to survival of untreated vs treated *Pcca*$^{-/-}$ mice. *P* values were calculated using a Log_rank (Mantel-Cox) test.**

Measurement of Plasma 2-MC in AAV9-hPCCA Treated *Pcca*$^{-/-}$ Mice

Animals dosed with AAV9-hPCCA were bled to measure a biomarker response (at day 30, 90 and

101), and then sacrificed to assess transgene expression and vector biodistribution. On day 30, the weights of the AAV9-hPCCA treated *Pcca*$^{-/-}$ mice were not different than treated control littermates (not presented). As illustrated in ***Figure 3***, treatment of *Pcca*$^{-/-}$ mice with AAV9-hPCCA resulted in a substantial reduction in the plasma levels of 2-MC as compared to the untreated animals at doses of 8.3 x10^{13} and 3.3 x10^{14} vg/ kg, with milder changes on day 30 in the lowest dose 8.3 x10^{12} vg/ kg cohort, and persisting in some treated mice to day 90, and beyond.

Figure 3. Plasma 2-MC Levels in AAV9-hPCCA Treated *Pcca*$^{-/-}$ Mice.

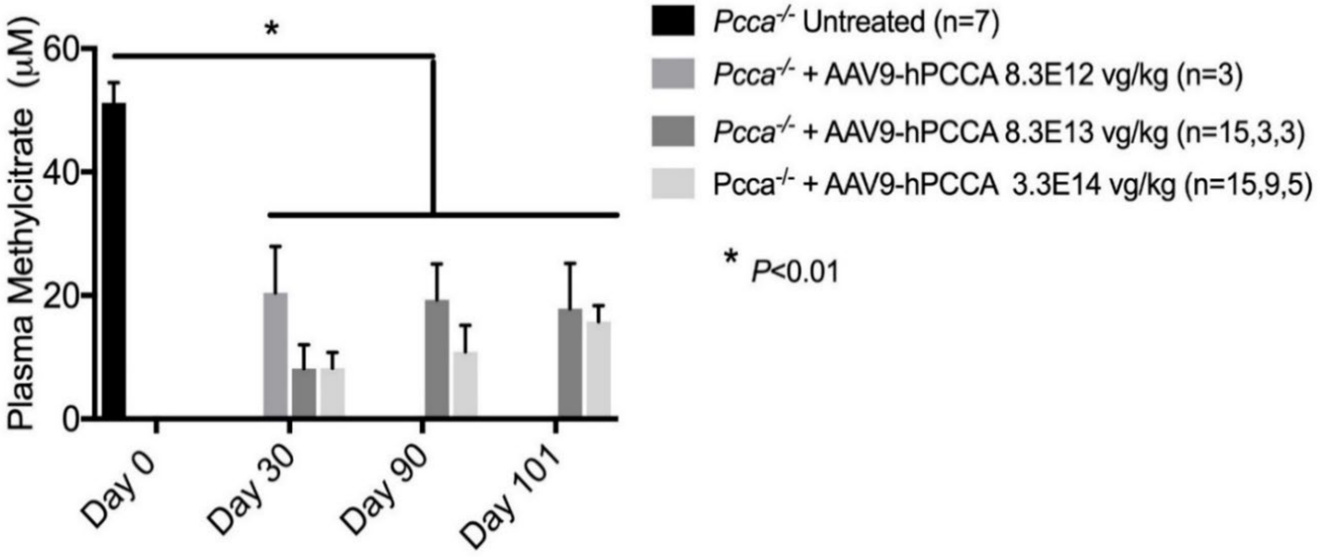

Mean ± S.D. of the plasma 2-MC levels in *Pcca*$^{-/-}$ mice treated at birth with AAV9-hPCCA. ****P*<0.01 for *Pcca*$^{-/-}$ treated vs untreated at the time of birth calculated by a two sided, two tailed t-test.

Transgene Expression in AAV9-hPCCA Treated *Pcca*$^{-/-}$ Mice

After treatment with AAV9-hPCCA, the transgene and protein levels were assessed in the liver of a small number of *Pcca*$^{-/-}$ mice treated with 8.3 x10^{13} vg/ kg or 3.3 x10^{14} vg/ kg of AAV9-hPCCA. The *PCCA* mRNA was quantified by RT-qPCR and the PCCA protein levels were measured by Western blotting at Day 30 and Day 101. The mRNA levels were normalized to endogenous glyceraldehyde 3-phosphate dehydrogenase (*Gapdh*) expression and protein levels were normalized to endogenous beta actin levels. The AAV encoded *PCCA* mRNA varied between ~15-25% of WT *Pcca* levels and the protein expression was at ~ 40% of the wild-type murine PCCA level (***Figure 4***).

Figure 4. Hepatic *PCCA* mRNA and PCCA Protein Expression in AAV9-hPCCA Treated *Pcca*$^{-/-}$ Mice

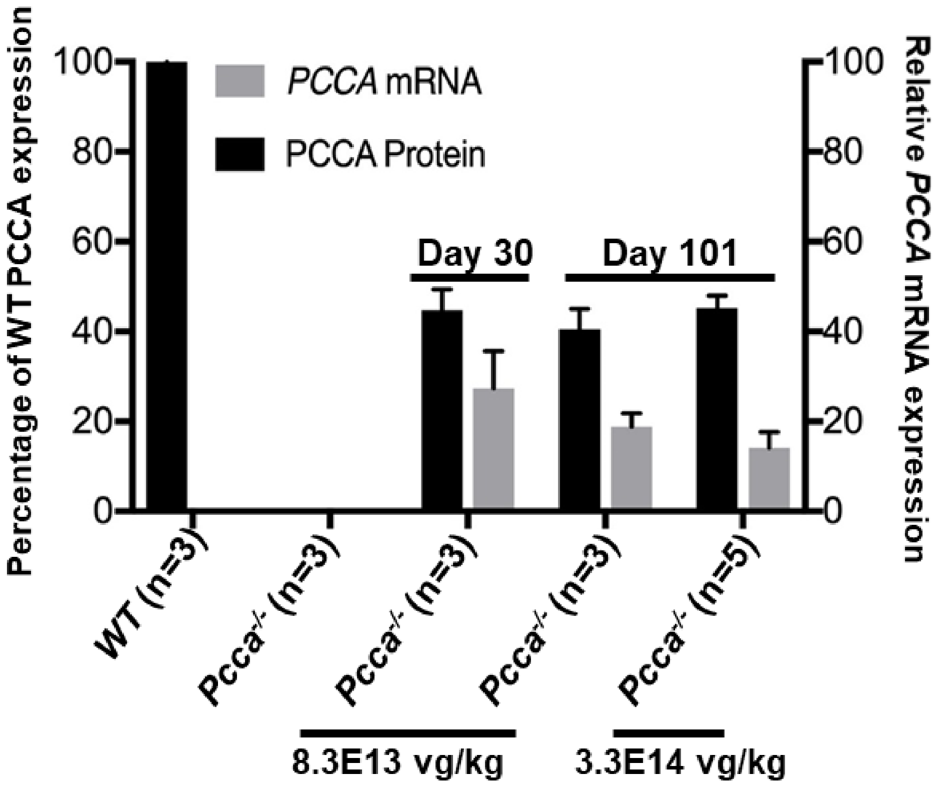

Quantitation (Mean ± S.D.) of *PCCA* mRNA and PCCA protein represented as a percentage of endogenous WT expression levels and normalized to beta-actin for protein levels and to *Gapdh* for mRNA expression.

Biodistribution in AAV9-hPCCA Treated Mice

In the PA patients, the target tissues are the liver and heart, and therefore, our initial survey was focused on hepatic and cardiac studies in mice treated with the AAV9-hPCCA vector. Using digital droplet (ddPCR) with probes designed to detect the *PCCA* transgene and the endogenous *Gapdh* locus, the copy numbers of the PCCA transgene were assessed at Day 30 and Day 101 in heterozygous *Pcca*$^{+/-}$ and homozygous *Pcca* $^{-/-}$ animals dosed with AAV9-hPCCA at 8.3 x10^{13} vg/ kg or 3.3 x10^{14} vg/ kg. As can be seen in ***Figure 5*** below, the AAV9-hPCCA genome is readily detected, indicating substantial transduction in the liver and heart in the *Pcca*$^{-/-}$ mice, with a better signal observed in the heart. Furthermore, the relative transduction between *Pcca*$^{-/-}$ mice and *Pcca*$^{+/-}$ littermate controls treated in an identical fashion appears similar.

Figure 5. Biodistribution of AAV9-hPCCA in Liver and Heart Tissues

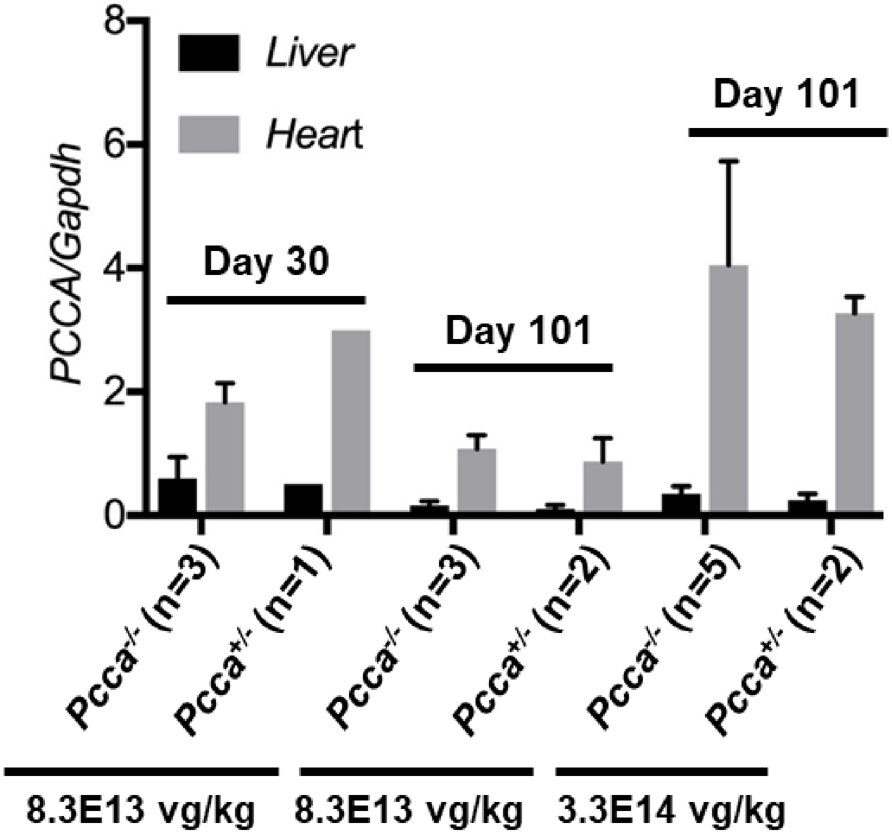

Vector copy number of the *PCCA* transgene in the liver and heart normalized to alleles of the *Gapdh* gene using ddPCR in the livers and hearts of AAV9-hPCCA treated *Pcca*$^{-/-}$ and *Pcca*$^{+/-}$ mice at 30- and 101-days post-treatment. Error bars are $\pm$ S.D.

2.1.4. Proposed GLP Toxicology Study

We are proposing a 6-month GLP toxicology and biodistribution study using GMP grade AAV9-hPCCA in 8 week old WT (C57BL/6) mice up to 1 x 10^{14} vg/kg via tail vein injection, to support the proposed peripheral IV or PICC line clinical ROA in the human study. Due to the need for immediate treatment, the POC studies have been done in neonatal *Pcca*$^{-/-}$ mice injected on P1 via the retro-orbital plexus (***Section 2.1.3***). Younger mice, perhaps equivalent in age to a human toddler (P7) cannot be injected by the tail vein, hence we are proposing to use juvenile mice so that the ROA might more closely mimic the ROA in our patient population. For the toxicology studies, WT mice will be used to assess toxicity of AAV9-hPCCA. Each group will consist of 10 Males/10 Females per timepoint and dose group (vehicle and three dose levels); endpoints will be evaluated at 1, 4, 12, and 24 weeks. The endpoints evaluated will include body weights, hematology, clinical chemistry, biodistribution, immunogenicity (anti-AAV9 capsid antibody) and histopathology (***Table 4***).

Table 4. GLP 6-Month Toxicity and Biodistribution Study Design

Group	Dose (vg/kg)	Number per time point (week 1, 4, 12, 24)	Subsets (5M/5F)	Endpoints	
				Tissue	Blood
1	0 (Vehicle control)	10M/10F	1-1	Biodistribution	Hematology<br>Biodistribution<br>Anti-AAV9 capsid antibody
			1-2	Histopathology	Clinical Chemistry
2	1×10^{13}	10M/10F	2-1	Biodistribution	Hematology<br>Biodistribution<br>Anti-AAV9 capsid antibody
			2-2	Histopathology	Clinical Chemistry
3	5×10^{13}	10M/10F	3-1	Biodistribution	Hematology<br>Biodistribution<br>Anti-AAV9 capsid antibody
			3-2	Histopathology	Clinical Chemistry
4	1×10^{14} or maximum feasible dose (MFD)	10M/10F	4-1	Biodistribution	Hematology<br>Biodistribution<br>Anti-AAV9 capsid antibody
			4-2	Histopathology	Clinical Chemistry

Note: All animals are 8 week old WT (C57BL/6) mice; ROA will be IV tail vein injection.

Study Outline

1. All animals will be dosed on week 8 with IV tail vein injection.
2. All animals will be observed twice daily during 1st week after dosing and at least once daily until study ends or more often as clinical signs warrant.
3. All animals will be weighed prior to the study for randomization purposes; approximately Day -3 to Day -1 and then weekly thereafter. Food consumption will be recorded weekly.
4. Biodistribution of tissue samples: Blood, injection site, heart, muscle, intestine, adipose tissue, brain, liver, lung, spleen, kidneys, and ovary/testis will be collected at each necropsy. Tissues from post-dose Day 7 animals in the control (Group 1) and high dose (Group 4) will be analyzed by qPCR and subsequently RT-PCR in positive tissues. Any tissues positive by qPCR will be tested in high dose groups at subsequent time points (weeks 4, 12, and 24).
5. Anti-AAV9 capsid antibodies will be evaluated at each necropsy timepoint using an Anti-

AAV9 capsid antibody assay developed and validated following FDA Guidance for Industry, "*Immunogenicity Testing of Therapeutic Protein Products - Developing and Validating Assays for Anti-Drug Antibody Detection*," January 2019.

6. Hematology and clinical chemistry from 5 mice/sex/group will be performed for all of the standard analytes, if feasible, as specified in the study table.
7. Histopathology: Slides from a standard list of tissues from animals in control (Groups 1) and high dose (Group 4) will be examined by the study pathologist at all necropsy timepoints. Any target tissues identified in high dose (Groups 4) will be examined in Groups 2 and 3.

2.2. Chemistry, Manufacturing and Controls

The investigational drug product consists of AAV9 vector expressing a functional human codon optimized *PCCA* cDNA under control of the *[a specific promoter]*, herein referred to as AAV9-hPCCA.

AAV9-hPCCA Vector Design

In humans, endogenous PCCA protein is ubiquitously expressed, therefore we designed a therapeutic transgene cassette with a constitutive promoter to enable wide expression and selected the AAV9 capsid to further enable hepatic and cardiac transduction. A schematic of the vector transgene and description of cassette features summarizing the salient features of the AAV vector is presented in ***Figure 6***.

Figure 6. A Schematic of the Vector Transgene and Description of the Cassette.

[Schematic of the vector and table containing length of different cassette elements]

For purposes of the GMP manufacturing process, the ITRs of the GMP grade AAV9-hPCCA have been optimized, strictly conserving all other elements of the research grade AAV9-hPCCA used in the POC studies, described under ***Section 2.1.3***.

AAV9-hPCCA will be manufactured using standard production methods for AAV9 vectors. To date, our contract manufacturing organization (CMO), Vigene Biosciences, Inc. (Vigene), has manufactured a small-scale feasibility batch (final report pending), and we project that Vigene will also manufacture the GMP grade investigational drug product. Vigene has demonstrated experience in the GMP manufacturing of investigational gene therapy products for clinical trials. We will provide the Agency all supporting manufacturing information in our planned pre-IND briefing package. Any preliminary CMC related guidance from the Agency would be appreciated.

2.3. Clinical

[The work related to the PA Natural History described in this section 2.3 has now been published elsewhere. For most updated results and information, please refer to the three articles below]

1. *Shchelochkov, O.A., et al. Severity modeling of propionic acidemia using clinical and laboratory biomarkers. Genet Med **23,** 1534–1542 (2021).*
2. *Shchelochkov O.A., et.al.. Chronic kidney disease in propionic acidemia. Genet Med. 2019 Dec;21(12):2830-2835. PMID: 31249402; PMCID: PMC7045176.*
3. *Shchelochkov, O.A., et al. Intellectual disability and autism in propionic acidemia: a biomarker-behavioral investigation implicating dysregulated mitochondrial biology. Mol Psychiatry **29**, 974–981 (2024).*

PA is a rare inborn error of metabolism resulting from deleterious variants in the *PCCA* or *PCCB* genes leading to impaired activity of PCC. PCC is a ubiquitously expressed mitochondrial enzyme whose function is closely linked to energy metabolism. The clinical course of PA is characterized by chronic multiorgan dysfunction punctuated by episodes of metabolic instability due to metabolic acidosis, ketonuria, hyperammonemia, and hypoglycemia leading to emergency room visits and hospitalizations (***Figure 7***). Most patients with PA present in the newborn period with poor feeding, vomiting, irritability and lethargy. Without treatment, these infants may develop neonatal encephalopathy, seizures, coma, and respiratory failure, which if left untreated can result in death. Universal newborn screening implemented in the United States (US) can identify most affected infants. Infrequently, newborn screening fails to identify affected newborns, usually infants homozygous for the "mild" Amish-Mennonite *PCCB* allele (*PCCB*:c.1606A>G, p.Asn536Asp). Such milder cases can present later in life with life-threatening dilated cardiomyopathy.

Figure 7. Clinical and Laboratory Manifestations of PA

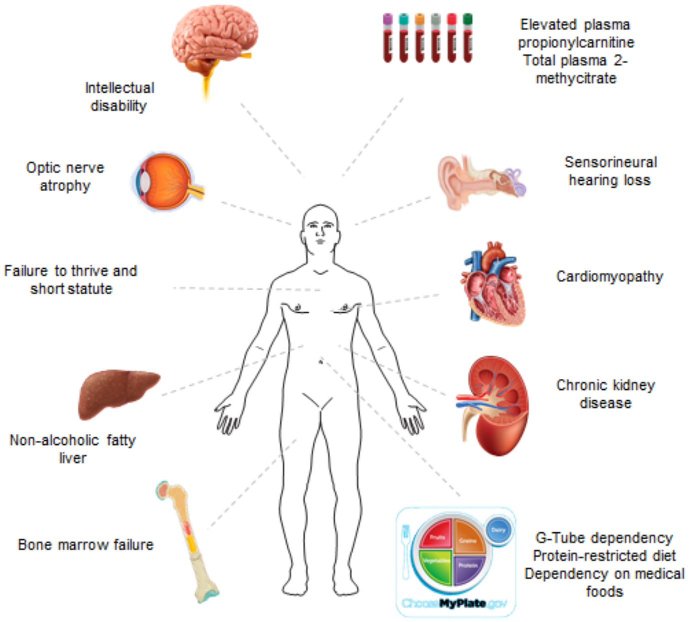

PA is a rare disorder with birth prevalence varying widely by region. Worldwide, the birth incidence has been reported as high as 1:1000 and as low as 1:500,000. The estimated birth incidence of PA in the US, depending on the region, ranges between 1:105,000 and 1:500,000 with the average birth incidence being ~1:243,000 (Chapman et al., 2018). Based on the annual US birth rate of 3.7M/year, approximately 15 children with *PCCA*- and *PCCB*-type PA are born in the US each year. Based on our good-faith assessment, we estimate there are ~50 *PCCA*-type PA patients living in the US.

There is no cure for PA. Current standards of care include strict adherence to low-protein diet with and without poorly palatable medical foods, supplementation with levocarnitine. Other treatments may apply if patients develop severe complications of PA, for example the use of cardiac medications in patients with cardiomyopathy or antiepileptic drugs in PA patients with epilepsy (Shchelochkov et al., 2012 [updated 2016, *[now updated 2024]*]). Some PA patients, who experience frequent hospitalizations due to metabolic instability and/or who develop dilated cardiomyopathy may undergo a liver or a combined liver-kidney transplantation and require life-long immunosuppression.

There is a lack of natural history studies longitudinally evaluating PA biomarkers and their association with outcomes corresponding to survival, functioning and disease burden. To close this gap, the Organic Acidemia Section of NHGRI initiated a natural history study of PA population (National Clinical Trial # ***NCT02890342***). The study is being conducted at the Clinical Center of NIH and has enrolled 40 participants ages 2 years and older (2.5 years – 54 years of age). The cohort features a genetically heterogeneous PA population, a high proportion of adult patients (~35%), and a subset of liver- and kidney-transplanted PA participants (***Figure 8***).

Figure 8. Clinical Characteristics of the NIH Natural History Cohort.

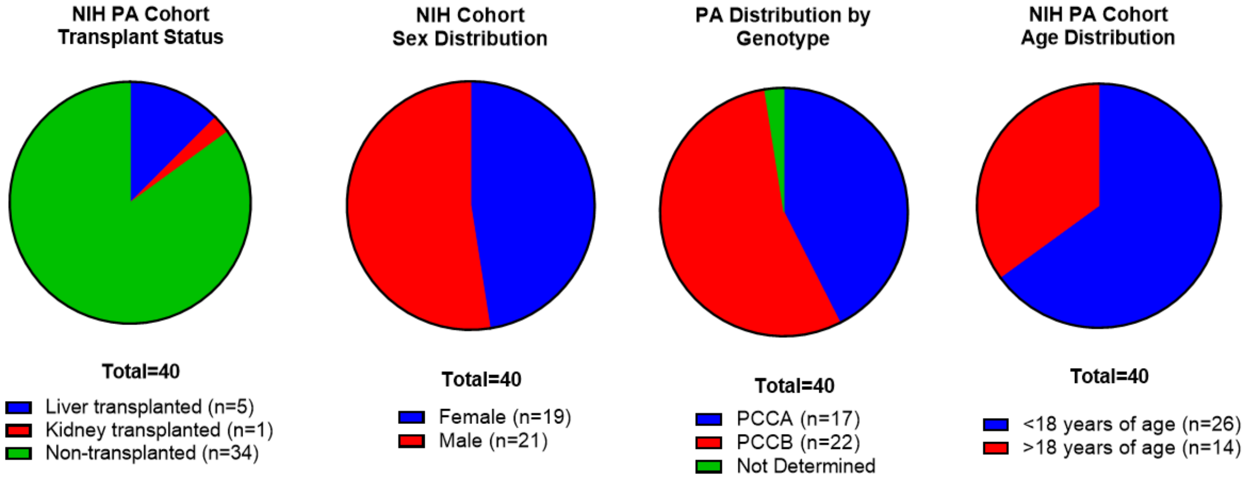

Cross-sectional and longitudinal studies of up to 500 clinical, laboratory, and imaging parameters in transplanted and non-transplanted participants in the NIH cohort reveals high prevalence of chronic complications including chronic kidney disease, *[another manifestation]* and optic nerve pathology, and sensorineural hearing loss (***Figure 9***).

Figure 9. Age-dependent Manifestations of PA Supporting Pediatric Use of Biologics Aiming to Restore Propionate Oxidation

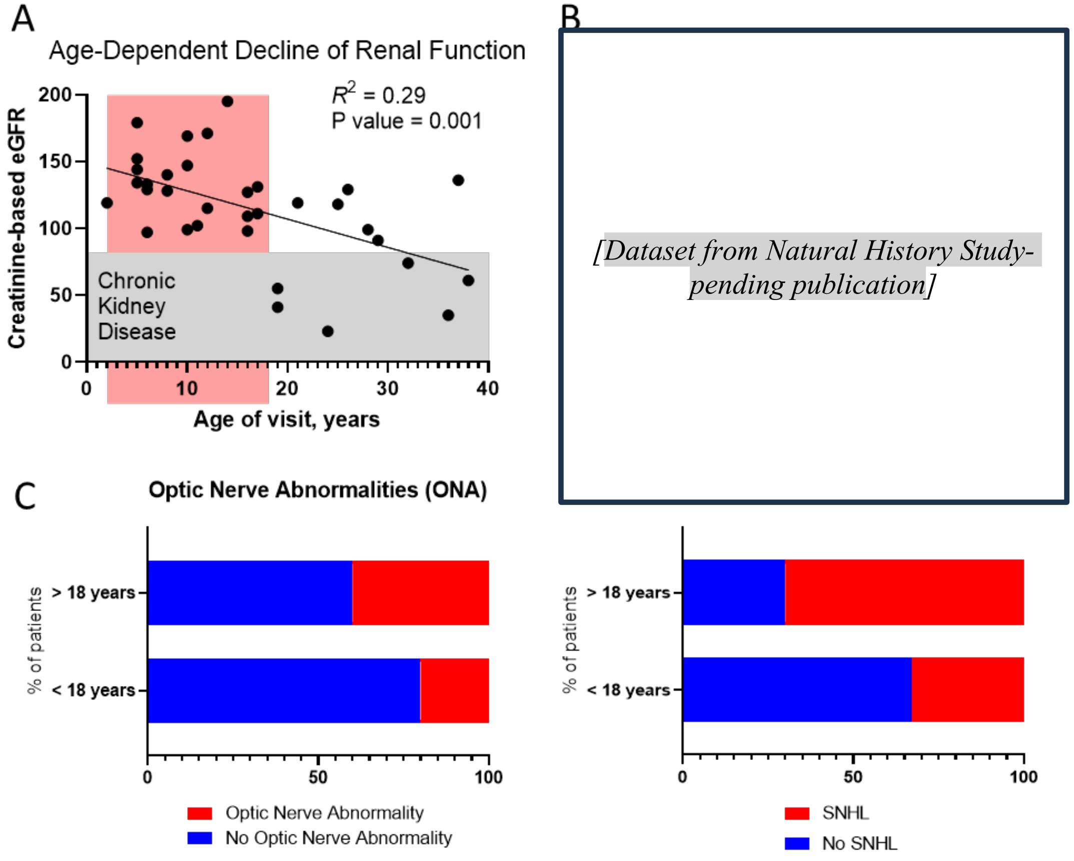

A-B. Data from the NIH natural history study cohort demonstrates an age-dependent decline of renal function and *[other manifestation]*. C-D. The prevalence of optic nerve abnormality and sensorineural hearing is higher in patients > 18 years of age. Abbreviations: eGFR- estimated glomerular filtration rate;; ONA optic nerve abnormality; SNHL - sensorineural hearing loss.

The NIH Natural History Study of PA has enabled identification of candidate pharmacodynamic/response biomarkers and candidate surrogate endpoints for PA (*Figure 10*). Specifically, we evaluated the performance of biomarkers relevant to PA: *in vivo* whole-body 1-^{13}C-propionate oxidation, plasma propionylcarnitine, plasma total 2-MC, creatinine and cystatin C-based estimated GFR in three contexts of use (diagnostic, pharmacokinetic/response, and monitoring). Using data obtained from non-transplanted and liver-transplanted PA participants, we evaluated performance of conventional diagnostic PA biomarkers (plasma total 2-MC and plasma propionylcarnitine) as candidate response biomarkers in the context of intervention aiming to restore

hepatic oxidation of propionate. We considered liver transplantation as a prototype for liver-directed biologics capable of improving propionic acid clearance. Our analysis revealed a statistical trend, but not a statistically significant decrease of plasma total 2-methylcitrate or plasma propionylcarnitine after liver transplant. The lack of biomarker response after liver transplantation can in part be explained by worsening of renal function after liver transplantation resulting in higher levels of plasma total 2-MC and plasma propionylcarnitine. In addition, we observed a positive correlation of plasma propionylcarnitine and dose of levocarnitine. These data suggest that while plasma total 2-MC and plasma propionylcarnitine can serve as response biomarkers, the interpretation of changes in their levels will depend on the dynamics of renal function and levocarnitine dosage after a therapeutic intervention. Also, these finding support the need to monitor renal function using creatinine-based and cystatin C-based eGFR as monitoring biomarkers.

Figure 10. Performance of PA Diagnostic Variables as Response Biomarkers Depends on the Renal Status and Levocarnitine Supplementation.

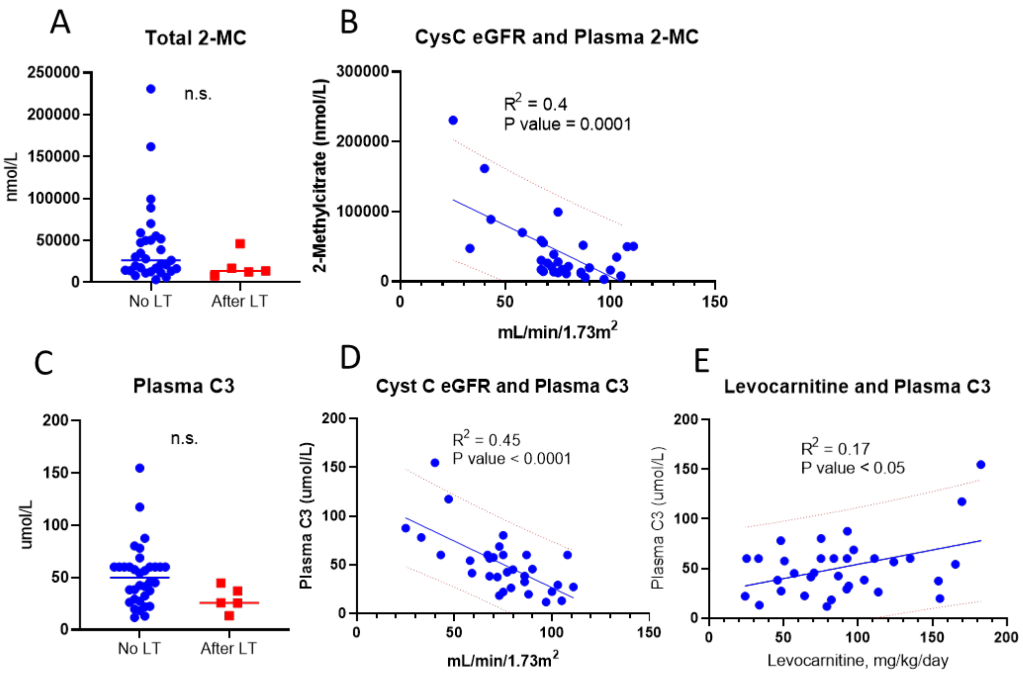

<u>A-B</u>. In the NIH natural history cohort of PA changes in plasma 2-MC were context specific. While there was a general trend towards lower 2-MC after liver transplant, the difference was not statistically significant. In part this was likely the result of worsening renal function reported in PA patients after are liver transplant as a result of exposure to immunosuppressive medications. <u>C-E.</u> A similar trend was observed for plasma C3. C3 was not significantly different after transplant and was associated with the decline of renal function and supplementation with oral levocarnitine. These finding motivated our search for other response biomarkers that would be less dependent on the renal function and medications. Abbreviations: 2-MC – 2-methylcitrate; CysC eGFR: cystatin C-based estimated glomerular filtration rate; LT: liver transplant; C3: propionylcarnitine

We sought to identify alternative biomarkers, which would be less dependent on the renal function and biochemically closely related to propionate metabolism. We established that *in vivo* 1-^{13}C-propionate oxidation (***Figure 11***) closely correlates with:

- established diagnostic biomarkers (plasma total 2-MC and plasma propionylcarnitine),
- clinically relevant outcomes (growth, intellectual disability, sensorineural hearing loss),
- does not depend on the renal function,
- and shows an unequivocal response to liver transplantation.

These data support the use of this novel test as a response biomarker in the context of AAV-mediated delivery of *PCCA*.

Figure 11. *In Vivo* 1-^{13}C-Propionate Oxidation Correlates with Diagnostic Biomarkers, Clinically Relevant Outcomes and Liver Transplant Status, and Does Not Depend on the Renal Function.

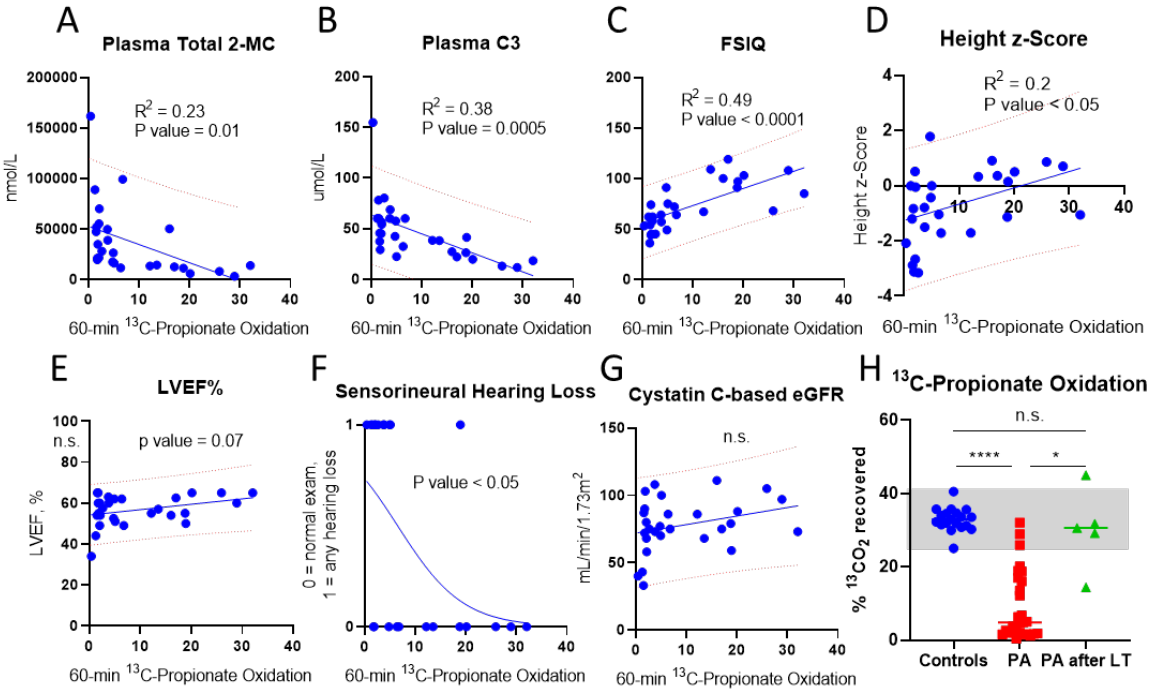

<u>A-F.</u> 1-^{13}C-Propionate Oxidation strongly correlated with laboratory parameters (plasma 2-MC, C3), and clinically relevant neurocognitive outcomes, linear growth, and sensorineural hearing loss. <u>G.</u> Importantly, there was no statistically significant association between propionate oxidation and estimated glomerular filtration rate. H. Propionate oxidation was significantly lower in PA patients compared to healthy controls and was restored to normal after liver transplantation. Abbreviations: 2-MC – 2-methylcitrate, C3 – propionylcarnitine, FSIQ – full-scale IQ, LVEF% - left ventricle ejection fraction, LT – liver transplant.

Based on the natural history data, we are proposing inclusion of PA patients aged 2-18 years in the FIH Phase 1/2 study because 1) PA patients manifest life-threatening debilitating illness in early childhood; 2) PA is poorly responsive to available medical therapy; 3) novel therapies are urgently

needed to prevent non-reversible disease complications; and 4) adult PA patients have high prevalence of severe kidney and heart disease, and irreversible disease complications to sensory organs making them high-risk subjects for study and less likely to benefit from gene therapy. We therefore propose the following clinical protocol to select and clinically manage participants enrolled in the study to evaluate the safety and efficacy of AAV9-hPCCA.

2.3.1. Proposed Phase 1/2 Clinical Trial Protocol Synopsis

[IMPORTANT NOTE: Clinical protocol design subject to change and descriptions below are not applicable for all diseases and studies]

**Title**	*An Open-Label Phase 1/2 Clinical Study of Adeno-Associated Virus Gene Delivery in Pediatric and Adult Patients with Propionic Acidemia Characterized by Genetic Defects in the Propionyl-CoA Carboxylase Subunit Alpha (PCCA) Gene*
**Sponsor**	NIH, NCATS
**Study Phase**	Phase 1/2
**Study Population**	Pediatric and adult patients, aged 2-18 years, with clinically, biochemically, and/or molecularly confirmed *PCCA* genetic mutations that cause PA.
**Number of Sites and Subjects**	One site; approximately 10 subjects.
**Treatment Groups**	1-3 patients in cohort 1 *[low dose]*, approximately 4 patients in cohort 2 *[medium dose]*, and approximately 4 patients in the optional cohort 3 *[high dose]*.
**Primary Objective**	To assess safety and tolerability of AAV9-hPCCA in patients with PA.
**Secondary Objectives**	To assess changes from baseline in response biomarkers after AAV9-hPCCA dosing in patients with *PCCA*-related PA.
**Study Design**	A phase 1/2, open-label safety, dose-escalation, dose-finding, single-center, single-dose, clinical study in subjects with *PCCA*-related PA.<br>The study will consist of the following 3 cohorts:<br>Cohort 1. 1-3 patients, *[low dose]*<br>Cohort 2. approximately 4 patients, *[medium dose]*<br>Cohort 3. approximately 4 patients, *[high dose]*<br><br>*Cohort 1. [low dose]*<br>At least 8 weeks will separate dosing between eligible study participants. A DSMB review of available safety data will commence at the end of 8 weeks after dosing of each patient. If the safety review is sufficient, dosing of the next patient will proceed. If clinical assessment and biomarker data do not support the efficacy of this dose in the first patient, we will proceed to cohort 2.<br>*Cohort 2.[medium dose]*<br>At least 8 weeks will separate dosing between eligible study participants. A DSMB review of available safety data will commence at the end of 8 weeks after dosing of each patient. If the safety review is sufficient, dosing of the next patient will proceed. If clinical assessment and biomarker

Clinical study design is subject to change and details herein are not applicable for all diseases/studies

Title	*An Open-Label Phase 1/2 Clinical Study of Adeno-Associated Virus Gene Delivery in Pediatric and Adult Patients with Propionic Acidemia Characterized by Genetic Defects in the Propionyl-CoA Carboxylase Subunit Alpha (PCCA) Gene*
	data do not support the efficacy of this dose, we will proceed to cohort 3.<br>*Cohort 3. [high dose]*.<br>The dosing of the high-dose cohort can commence 8 weeks after dosing the last patient in cohort 2, if the safety data review by DSMB is sufficient.<br><br>***Study timeline and schedule of events (Appendix B)*:**<br>Study candidates will be evaluated for study eligibility as part of the screening evaluation 1 month prior to the anticipated dosing day. Patients may co-enroll in the Natural History Study of propionic acidemia conducted at NIH. Patients will undergo collection of labs, diagnostic studies, dietary assessment, neurocognitive evaluation to determine their eligibility according to the inclusion/exclusion criteria outlined in this clinical synopsis.<br>Patients will undergo a non-invasive *in vivo* study of propionate oxidation within 1 month prior to the anticipated dosing day.<br>Patients who meet the eligibility criteria during the screening will be re-evaluated 2 days prior to the anticipated infusion date to ensure they continue meeting the eligibility criteria.<br>Patients eligible to participate will commence prophylactic treatment with oral corticosteroid (prednisone or prednisolone): 1 mg/kg/day (not to exceed a maximum dose of 60 mg/day) started 24±4 hours prior to starting administration of AAV9-hPCCA. This dose will continue for 30 days but could be extended for another 2 weeks if there is any evidence of the immunological response to AAV9. Upon completion of the oral 1 mg/kg/day corticosteroid course, the corticosteroid dose will be tapered over the following 28 days.<br>Patients will be hospitalized for the administration of AAV9-hPCCA 2 days prior to the anticipated dosing day. Patients will be observed at the hospital for 72 hours post-dosing prior to their discharge to home. The total period of observation surrounding the infusion will be 5 days (-2 days and 3 days after infusion).<br>Following the infusion of AAV9-hPCCA patients will be evaluated weekly in the first month, then every other week in Months 2 and 3, then at Months 6, 9, and 12.<br>During the short-term 12-month follow-up study, patients will continue taking their baseline medications and diet as advised by their healthcare providers, unless a change is warranted based on the clinical findings.<br><br>*The long-term 5-year study*.<br>Patients who completed the 12-month short-term study will roll into the long-term study, which will include interval assessment of safety and efficacy of AAV9-hPCCA for up to 5 years of follow-up.<br>*Rules for suspending the study*:<br>The study will be *suspended* if within the first 12 months after the administration of AAV9-hPCCA, any of the following occurs:<br>1. A patient dies.<br>2. A patient develops a malignancy.<br>3. A patient experiences a grade 4 or higher adverse event deemed to be related to AAV9-hPCCA.<br>4. Any grade 4 serious adverse event that could be related to study procedures (e.g., complications

Clinical study design is subject to change and details herein are not applicable for all diseases/studies

**Title**	*An Open-Label Phase 1/2 Clinical Study of Adeno-Associated Virus Gene Delivery in Pediatric and Adult Patients with Propionic Acidemia Characterized by Genetic Defects in the Propionyl-CoA Carboxylase Subunit Alpha (PCCA) Gene*
	from corticosteroids or stable isotope administration).<br>5. The DSMB chair or sponsor of the study determines that a medical event requires additional evaluation by the full DSMB.<br>*Rules for stopping the study*<br>The study will be *stopped* if within the first 12 months after the administration of AAV9-hPCCA, if any of the following occurs:<br>1. The sponsor or FDA determines that an event or data warrant termination of the study for any reason.<br>2. A patient develops malignancy determined by the sponsor or FDA to be related to the AAV9-hPCCA administration.<br>3. A patient death is determined by the sponsor or FDA to be related to the AAV9-hPCCA administration.<br>If the study has been stopped for any reason listed above, patients who had been dosed prior to the qualifying event, will continue to be followed as part of the long-term 5-year follow-up study.
**Estimated Study Duration**	13 months, including screening and baseline assessments -1 month before the dose administration, hospitalization at –2 day before the dose administration, post-infusion observation for 3 days, 1-year follow-up after the dose administration.<br>Upon completion of the study, participants will enter a 5-year follow-up study.
**Summary of Subject Eligibility Criteria**	*Inclusion Criteria*:<br>1. Age 2-18 years of age at the time of consent (whenever applicable, assent will be obtained).<br>2. Ability and willingness to comply with the scheduled study visits and procedures.<br>3. Complete vaccination according to the CDC vaccination schedule for age at the time of consent, with exception of live attenuated vaccines with appropriate time intervals post-immunization per accepted recommendations.<br>4. Males and females with the diagnosis of *PCCA*-related PA confirmed clinically, biochemically, and molecularly.<br>5. No episodes of metabolic decompensation for 2 months prior to the scheduled dose administration.<br>6. Females of childbearing potential who are sexually active must use at least one method of contraception.<br>*Exclusion Criteria*:<br>1. Males and females with the diagnosis of *PCCB*-related PA confirmed clinically, biochemically, and molecularly.<br>2. History of the following interventions at any point in the past:<br>a. Gene therapy<br>b. Solid organ transplantation<br>c. Cell transfer therapy<br>d. History of malignancy<br>3. History of investigational drugs within 5 half-lives of the drug or within 28 days before the first screening visit, whichever comes first.

Clinical study design is subject to change and details herein are not applicable for all diseases/studies

**Title**	*An Open-Label Phase 1/2 Clinical Study of Adeno-Associated Virus Gene Delivery in Pediatric and Adult Patients with Propionic Acidemia Characterized by Genetic Defects in the Propionyl-CoA Carboxylase Subunit Alpha (PCCA) Gene*
	4. History of immunomodulating drugs (e.g., corticosteroids or IVIG) withing 3 months before the first screening visit.<br>5. Anti-AAV9 neutralizing antibody titer above *[the cut-off]*<br>6. Left ventricular ejection fraction *[XX]* by transthoracic ECHO or other comparable diagnostic modality.<br>7. QTcB *[XX]* ms for either male or female participants.<br>8. Creatinine-based estimated GFR of *[XX]* or ongoing dialysis for chronic kidney disease.<br>9. Any of the following laboratory finding at the time of the first screening visit:<br>a. Hemoglobin *[XX]*<br>b. Absolute neutrophil count *[XX]*<br>c. Platelet count *[XX]*<br>d. ALT, AST, or total bilirubin *[XX]* times the upper normal limit for age<br>e. Plasma lipase or amylase*[XX]* times the upper normal limit for age<br>f. Plasma ammonia *[XX]* times the upper normal limit for age or clinical symptoms of hyperammonemia (e.g., lethargy, excessive irritability, vomiting)<br>g. Alpha-fetoprotein *[XX]* times the upper normal limit for age<br>10. History of anaphylaxis.<br>11. History of severe allergic reactions to any components of the gene therapy product.<br>12. Pregnant or breast-feeding.<br>13. History of a medical condition or family history of a disorder (e.g., a familial cancer predisposition syndrome) which, in the opinion of investigator, can exclude a patient from participating in the study.
**Concomitant Medications**	Oral corticosteroid (prednisone or prednisolone): 1 mg/kg/day (not to exceed a maximum dose of 60 mg/day) started 24±4 hours prior to starting AAV9-hPCCA. This dose will continue for 30 days but could be extended for another 2 weeks if there is any evidence of the immunological response to AAV. Upon completion of the oral 1 mg/kg/day corticosteroid course, the dose will be tapered over the next 28 days.<br>Sodium 1-^{13}C-propionate is used as a non-radioactive tracer to measure the activity of propionate oxidation before and after infusion of AAV9-hPCCA. Sodium 1-^{13}C-propionate dosed as *[XX]* mg/kg/dose will be given orally or via G-tube (J-tube) per study.
**Drug, Drug Dosage, and Formulation**	AAV9-hPCCA is non-pyrogenic solution that will be stored per manufacturer's specifications until ready to be used. It will be thawed prior to administration as a one-time IV infusion given over *[XX]* minutes using a syringe pump at a rate of no more than *[XX]* ml/kg/hour.
**Control Group**	Baseline and historically controlled. Historical control from the NIH natural history study of PA.
**Dose Justification**	Initial FIH dose and dose-escalation plan calculated from pre-clinical AAV dosing studies in the neonatal knock out mouse model of PA caused by deleterious variants in *PCCA*.

Clinical study design is subject to change and details herein are not applicable for all diseases/studies

**Title**	*An Open-Label Phase 1/2 Clinical Study of Adeno-Associated Virus Gene Delivery in Pediatric and Adult Patients with Propionic Acidemia Characterized by Genetic Defects in the Propionyl-CoA Carboxylase Subunit Alpha (PCCA) Gene*
**Route of Administration**	A single peripheral IV infusion or in participants with poor or unreliable peripheral IV access, a peripherally inserted central catheter (510k-cleared PICC line) will be used.
**Dose Escalation**	The study is composed of cohort 1*[low dose]*, cohort 2 *[medium dose]*, and a cohort 3 *[high dose]*
**Procedures**	An *in vivo* 1-^{13}C-propionate oxidation study.<br>Peripheral IV insertion or ultrasound-guided PICC insertion at Day -2
**Primary Endpoint**	Incidence of treatment-related adverse events, treatment-emergent adverse events, and serious adverse events for each cohort.
**Secondary Endpoint**	Absolute and percent change*[two disease specific biomarkers]*
**Exploratory Endpoints**	*[Disease specific exploratory endpoints included]*
**Statistical Considerations**	Descriptive statistics

2.4. PaVe-GT Platform Program

Platform Vector Gene Therapy (PaVe-GT) is an NIH program whose main goal is to test whether the efficiency of gene therapy development and clinical testing can be increased by standardizing, where applicable, pre-clinical processes for four different rare disease gene targets. PaVe-GT will use the same AAV serotype, AAV9, as a platform vector to develop gene therapy products for four very low prevalence orphan diseases. The rare monogenic diseases selected include two organic acidemias and two congenital myasthenic syndromes. The organic acidemias under study are propionic acidemia (caused by PCCA deficiency) and isolated methylmalonic acidemia (MMAB deficiency/cobalamin type B methylmalonic acidemia). The congenital myasthenic syndromes under study are characterized by deficiency of the protein DOK7 (downstream of tyrosine kinase 7) or deficiency of Collagen Q (ColQ), a specific nonfibrillar collagen.

The AAV9 vector was selected for the PaVe-GT program because of its broad cell and tissue tropism, including the liver, muscle, and CNS. The AAV9 vector used in clinical trials here will be produced in the same manufacturing facility, using the same production and purification methods, with the only difference being the therapeutic gene constructs. While POC studies will be conducted individually for the selected disorders, we hypothesize that additional efficiencies will be gained in the biodistribution studies and potentially, toxicology, as the preclinical testing advances.

NCATS held an informational meeting with FDA on July 2, 2019, regarding the development of the Adeno-Associated Virus 9 (AAV9) PaVe-GT program, established for the treatment of rare diseases using gene therapies (***please see attachment "Supplemental Information"***), where FDA was

Clinical study design is subject to change and details herein are not applicable for all diseases/studies

supportive of the NCATS development of a rare disease platform vector gene therapy approach. Furthermore, FDA was supportive in providing advice and disseminating information to the public, in particular the rare disease community, when appropriate.

NCATS and FDA discussed streamlining the platform-based approach during the July 2, 2019, meeting. FDA indicated that as per FDA Guidance for Industry, "*Preclinical Assessment of Investigational Cellular and Gene Therapy Products*", November 2013, biodistribution studies will not be required for every gene target being developed using the PaVe-GT platform, and that a total of one biodistribution study for the platform should be sufficient. Furthermore, as discussed during the July 2, 2019, meeting, NCATS would like to remind the Agency of its intent to make interactions with and FDA feedback/guidance publicly available, including meeting summaries, as deemed appropriate. NCATS believes that the public dissemination of regulatory knowledge gained from the PaVe-GT platform will be of tremendous value to the orphan disease community in helping to develop potential lifesaving gene therapies.

Regarding the PaVe-GT platform program vector manufacturing process, NCATS is planning to submit and maintain all AAV9 vector related manufacturing information under the AAV9-hPCCA IND and cross-reference this information for all subsequent gene target INDs utilizing the PaVe-GT platform.

2.5. Planning for Expedited Pathways

PA is a rare inborn error of metabolism resulting from deleterious variants in the *PCCA* or *PCCB* genes leading to impaired activity of PCC. PCC is a ubiquitously expressed mitochondrial enzyme whose function is closely linked to energy metabolism (***Section 2.3***). PA is a serious and life-threatening disease for which there are no satisfactory or approved therapies, and for which there are substantial unmet medical needs for PA patients.

Fast Track Designation

A substantial amount of non-clinical data has been generated in support of AAV9-hPPCA efficacy in the *Pcca* $^{-/-}$ neonatal knock-out mouse model, representing the severe form of PA disease progression in humans (***Section 2.1***). As per FDA Guidance for Industry, "*Expedited Programs for Regenerative Medicine Therapies for Serious Conditions*," February 2019, and FDA's clarification of FT designation criteria, "*An investigational new drug that is intended to treat a serious condition, and for which nonclinical or clinical data demonstrate the potential to address an unmet medical need in patients with such condition, can receive fast track designation*" (bold underline added) we intend to utilize the nonclinical data generated from the *Pcca* $^{-/-}$ neonatal knock-out mice in support of a request for FT designation, at the time of original IND submission.

Regenerative Medicine Advanced Therapy (RMAT) Designation

We have collected data utilizing cross-sectional and longitudinal studies of up to 500 clinical, laboratory, and imaging parameters in transplanted and non-transplanted participants that have

enabled identification of candidate pharmacodynamic/response biomarkers and candidate surrogate endpoints for PA. Specifically, we evaluated the performance of clinical biomarkers relevant to PA: *in vivo* whole-body 1-^{13}C-propionate oxidation, plasma propionylcarnitine, C3/C2 ratio, plasma total 2-MC, creatinine and cystatin C-based estimated GFR in three contexts of use (diagnostic, pharmacokinetic/response, and monitoring).

As per FDA Guidance for Industry, "*Expedited Programs for Regenerative Medicine Therapies for Serious Conditions*," February 2019, and FDA's clarification of RMAT designation, "*It meets the definition of regenerative medicine therapy; It is intended to treat, modify, reverse, or cure a serious condition; and preliminary clinical evidence indicates that the regenerative medicine therapy has the potential to address unmet medical needs for such condition*" (bold underline added). we intend to submit a request for RMAT designation, pending our anticipated efficacy outcome of data collected through our clinical response biomarkers, 1-2 months post AAV9-hPCCA treatment initiation in the first cohort of PA patients under the proposed clinical protocol (***Section 2.3***). We believe that any initial efficacy signal observed in these biomarkers would be indicative of preliminary clinical evidence in support of RMAT designation.

3. REFERENCES

1) Brooks, P.J., Ottinger, E.A., Portero, D., Lomash, R.M., Alimardanov, A., Terse, P., Xu, X., Chandler, R.J., Geist Hauserman, J., Esposito, E., *et al.* (2020). The Platform Vector Gene Therapies Project: Increasing the Efficiency of Adeno-Associated Virus Gene Therapy Clinical Trial Startup. Hum Gene Ther *31*, 1034-1042.
2) Chandler, R.J., Chandrasekaran, S., Carrillo-Carrasco, N., Senac, J.S., Hofherr, S.E., Barry, M.A., and Venditti, C.P. (2011). Adeno-associated virus serotype 8 gene transfer rescues a neonatal lethal murine model of propionic acidemia. Hum Gene Ther *22*, 477-481.
3) Chapman, K.A., Gramer, G., Viall, S., and Summar, M.L. (2018). Incidence of maple syrup urine disease, propionic acidemia, and methylmalonic aciduria from newborn screening data. Mol Genet Metab Rep *15*, 106-109.
4) Guenzel, A.J., Collard, R., Kraus, J.P., Matern, D., and Barry, M.A. (2015). Long-term sex-biased correction of circulating propionic acidemia disease markers by adeno-associated virus vectors. Hum Gene Ther *26*, 153-160.
5) Guenzel, A.J., Hillestad, M.L., Matern, D., and Barry, M.A. (2014). Effects of adeno-associated virus serotype and tissue-specific expression on circulating biomarkers of propionic acidemia. Hum Gene Ther *25*, 837-843.
6) Guenzel, A.J., Hofherr, S.E., Hillestad, M., Barry, M., Weaver, E., Venezia, S., Kraus, J.P., Matern, D., and Barry, M.A. (2013). Generation of a hypomorphic model of propionic acidemia amenable to gene therapy testing. Mol Ther *21*, 1316-1323.
7) Hofherr, S.E., Senac, J.S., Chen, C.Y., Palmer, D.J., Ng, P., and Barry, M.A. (2009). Short-term rescue of neonatal lethality in a mouse model of propionic acidemia by gene therapy. Hum Gene Ther *20*, 169-180.
8) Miyazaki, T., Ohura, T., Kobayashi, M., Shigematsu, Y., Yamaguchi, S., Suzuki, Y., Hata, I., Aoki, Y., Yang, X., Minjares, C., *et al.* (2001). Fatal propionic acidemia in mice lacking propionyl-CoA carboxylase and its rescue by postnatal, liver-specific supplementation via a transgene. J Biol Chem *276*, 35995-35999.
9) Shchelochkov, O.A., Carrillo, N., and Venditti, C. (2012 [updated 2016] *[now updated 2024]*]). Propionic Acidemia. In GeneReviews((R)) [Internet], pp. 1-30.
10) Surtees, R.A., Matthews, E.E., and Leonard, J.V. (1992). Neurologic outcome of propionic acidemia. Pediatr Neurol *8*, 333-337.

APPENDIX A

(List of all AAV9-hPCCA Treated Mice)

Genotype	Treatment	Dose (vg/pup)	Dose (vg/kg)	Sex	Number of Mice
Pcca$^{-/-}$	None	N/A	N/A	Not Available	24
Pcca$^{+/-}$	None	N/A	N/A	M	6
Pcca$^{+/-}$	None	N/A	N/A	F	11
Not Available	None	N/A	N/A	Not Available	12
Pcca$^{-/-}$	PBS	N/A	N/A	Not Available	11
Pcca$^{+/-}$	PBS	N/A	N/A	M	4
Pcca$^{+/-}$	PBS	N/A	N/A	F	5
Not Available	PBS	N/A	N/A	Not Available	4
Pcca$^{-/-}$	AAV9-hPCCA	1 x10^{10}	8.3 x10^{12}	M	1
Pcca$^{-/-}$	AAV9-hPCCA	1 x10^{10}	8.3 x10^{12}	F	2
Pcca$^{-/-}$	AAV9-hPCCA	1 x10^{10}	8.3 x10^{12}	Not Available	1
Pcca$^{+/-}$	AAV9-hPCCA	1 x10^{10}	8.3 x10^{12}	M	4
Pcca$^{+/-}$	AAV9-hPCCA	1 x10^{10}	8.3 x10^{12}	F	7
Not Available	AAV9-hPCCA	1 x10^{10}	8.3 x10^{12}	Not Available	7
Pcca$^{-/-}$	AAV9-hPCCA	1 x10^{11}	8.3 x10^{13}	M	11
Pcca$^{-/-}$	AAV9-hPCCA	1 x10^{11}	8.3 x10^{13}	F	13
Pcca$^{-/-}$	AAV9-hPCCA	1 x10^{11}	8.3 x10^{13}	Not Available	7
Pcca$^{+/-}$	AAV9-hPCCA	1 x10^{11}	8.3 x10^{13}	M	20
Pcca$^{+/-}$	AAV9-hPCCA	1 x10^{11}	8.3 x10^{13}	F	17
Not Available	AAV9-hPCCA	1 x10^{11}	8.3 x10^{13}	Not Available	12
Pcca$^{-/-}$	AAV9-hPCCA	4 x10^{11}	3.3 x10^{14}	M	4
Pcca$^{-/-}$	AAV9-hPCCA	4 x10^{11}	3.3 x10^{14}	F	8
Pcca$^{+/-}$	AAV9-hPCCA	4 x10^{11}	3.3 x10^{14}	M	2
Pcca$^{+/-}$	AAV9-hPCCA	4 x10^{11}	3.3 x10^{14}	F	3
Not Available	AAV9-hPCCA	4 x10^{11}	3.3 x10^{14}	Not Available	1
				Total# of mice=	197

Note: *Pcca*$^{-/-}$ (Homozygous knockout); *Pcca*$^{+/-}$ (Heterozygous animals exhibiting a WT phenotype). The genotypes and sexes for mice not found after injection or for mice genotype but died before the sex could be established are shown as not available.

APPENDIX B

[Schematic of study timeline and schedule of activities table]

INTERACT Meeting Comments

Our Reference: [*Application reference number, provided by FDA*]

DATE: July 13, 2021 **PAGES:** # 24

TO: [*Name of NCATS contact]*
National Center for Advancing Translational Sciences (NCATS)
9800 Medical Center Dr.
Rockville, MD 20850

FROM: [*Name of FDA contact*]
Regulatory Project Manager
Division of Regulatory Project Manager
Office of Tissues and Advanced Therapies

SUBJECT: To discuss the investigational gene therapy product, AAV9-hPCCA, intended for the treatment of pediatric and adult patients, aged 2-18 years, with Propionic Acidemia (PA) resulting from a deficiency of Propionyl-CoA Carboxylase, alpha subunit (PCCA).

PRODUCT: Adeno-Associated Virus 9 vector expressing a functional human codon optimized cDNA encoding the PCCA, under control of the [*specific promoter*].

PROPOSED INDICATION: Treatment of pediatric and adult patients, aged 2-18 years, with PA resulting from a deficiency of PCCA.

FDA Participants: [*List of FDA participants*]

U.S. Food & Drug Administration
10903 New Hampshire Avenue
Silver Spring, MD 20993
www.fda.gov

This material consists of our INTERACT meeting comments in response to your questions and any additional comments in preparation for the discussion at the teleconference meeting scheduled for July 14, 2021, 11AM-12PM. We are sharing this material to promote a collaborative and successful discussion at the meeting.

If you determine that discussion is needed for only some of the original questions, you have the option of reducing the agenda. If you have questions regarding specific comments or advice, please inform us so that the appropriate members of the Review Committee can provide clarification during the reserved meeting time. The meeting discussion will be limited to the initial questions submitted in your meeting package or to questions regarding our comments. CBER advice given during INTERACT meetings is informal and non-binding. Therefore, official meeting minutes will not be issued.

Please include a reference to [*application reference number*] in your future submissions related to the subject product.

INTERACT Meeting Comments

Sponsor Question 1:

Does the Agency agree that the efficacy observed in the proof of concept (POC) studies in the Pcca-/- neonatal mouse model (Section 2.1.3) is sufficient to support proposed clinical testing?

CBER Response to Question 1:

Your proposed first-in-human (FIH) clinical trial will enroll individuals with an age range from 2 to 18 years old. This trial represents more than a minor increase over minimal risk. Therefore, per 21 CFR 50.52, prior to initiating a study in children, in accordance with Subpart D, you must provide evidence that administration of the study agent provides a prospect of direct benefit (PDB). Based on the information provided in Section 2.13 (pages 16-21) of your INTERACT meeting package, we tentatively agree that the summarized data generated following retro-orbital injection of AAV9-hPCCA in the *Pcca-/-* neonatal mice are sufficient to support a PDB. However, please note that a final determination as to the adequacy of these data will be made following our review of comprehensive material, including complete study reports, that you should provide in your IND. Please also address the following comments in your pre-IND and IND submissions:

1. Please provide a comprehensive discussion, with accompanying data, of the biological relevancy of the *Pcca-/-* neonatal mouse model to the proposed patient population, including: a) progression of the abnormal phenotype observed in this model (i.e., biochemical, morphological, functional), b) the similarities and differences in this model and the disease phenotype (e.g., pathophysiology, biochemistry, functional changes, etc.), and c) the timing of AAV9-hPCCA

administration in this model relative to the disease status in the proposed patient population.

2. Please provide a summary table with the similarities and differences between the preclinical lot(s) of AAV9-hPCCA administered to the mice compared to the clinical lot(s).

3. Please confirm that the assay that will be used to determine the vector titers for the clinical lots of AAV9-hPCCA are also used for the lots administered in the POC study. If the same assay was not used, the vector titer for the preclinical lot(s) should be re-determined using the planned clinical assay and this result should be used to re-calculate the vector dose levels administered in your POC study.

4. Please provide data from bench testing that confirm the compatibility of the preclinical lot(s) of AAV9-hPCCA with the vector delivery system (e.g., needle/syringe system) used in the POC study. Your evaluation should include the ability to consistently deliver accurate pre-specified dose levels of AAV9-hPCCA (e.g., assessment of vector genomes after passage of the preclinical lot through the needle/syringe delivery system used in the preclinical studies). If vector loss is observed, please provide, in the study report and in data tables, the actual vector dose level administered.

5. You described (Appendix A, page 37) that *Pcca+/-* mice exhibit a wild type phenotype; and genotypes and sexes for mice were not found after injection or not available for those that died before genotype and sex could be established. Please confirm that you only included *Pcca-/-* neonatal mice in the study (Table 1, page 16).

6. Please specify a) the method used to randomly assign animals to all study groups, and b) the method for staggering dosing of animals. Please discuss the effect of these study design elements on the potential for study bias.

7. Please explain the procedure used to collect blood from the mice at multiple intervals (Days 30, 90, and 101 post-dose) for plasma levels of 2-methylcitrate (2-MC) and provide the determination method for 2-MC levels. Please confirm that the plasma 2-MC levels depicted in Figure 3 (page 19) represent the mean ±SD from individual animals, and not from pooled samples for each group.

Sponsor Question 2:

Does the agency agree with our proposal of establishing the FIH dosing based on the Pcca-/- neonatal mouse model studies (Section 2.1.3)?

CBER Response to Question 2:

We cannot yet agree to your proposal to establish the clinical starting dose level based only on the POC data in the *Pcca*-/- neonatal mouse model provided in

Section 2.1.3. These POC data in combination with the safety data from the GLP toxicology study should inform the starting dose level for your planned FIH clinical trial.

Sponsor Question 3a:

Does the Agency agree with the proposed toxicology study design in WT mice, specifically:

a. Does the Agency agree with the proposed toxicology dose of up to 1 x 1014 vg/kg in support of the proposed FIH doses?

CBER Response to Question 3.a:

Discussion of definitive toxicology study designs is outside the scope of an INTERACT meeting. Please refer to CBER's SOPP 8214, titled 'INTERACT Meetings with Sponsors for Drugs and Biological Products' at (https://www.fda.gov/media/124044/download) for the details. Thus, we recommend that you refrain from initiating this study until you receive our feedback in a future pre-IND meeting. We have the following general comments regarding this study. However, we emphasize that these comments are not all-inclusive, and that you should provide a detailed study protocol in your pre-IND submission.

1. The lot(s) of AAV9-hPCCA administered in this definitive study should be identical to the intended clinical product in terms of manufacturing process, product identity, and final formulation.

2. Please note that the POC data provided in Section 2.1.3 (pages 16-21) show that, following AAV9-hPCCA IV administration, *Pcca*-/- neonatal mice have a dose-dependent improvement of the disease phenotype, including increased survival and reduction of plasma 2-MC levels, with the highest dose level of 3.3 x 10^{14} vg/kg resulting in the most significant benefit. Please note that the dose levels specified in Table 4 (page 22) for your toxicology study will not support administration of AAV9-hPCCA at a clinical dose level higher than 1 x 10^{14} vg/kg.

Sponsor Question 3b:

b. Does the Agency agree that the GLP toxicology study ROA, tail vain injection in 8 week WT mice, is sufficient to support our proposed FIH human trial using either the IV or PICC line ROA?

CBER Response to Question 3.b:

The proposed clinical route of administration is via intravenous (IV) injection. Thus, IV administration of AAV9-hPCCA in the GLP toxicology study is appropriate. In your pre-IND and IND submissions, please provide an explanation regarding the age of the wild-type mice at the time of dosing.

We acknowledge that you plan to include the peripherally inserted central catheter (PICC) route of administration in your clinical trial if peripheral IV access in a subject is poor or unreliable (page 33). Please provide a table that compares administration of AAV9-hPCCA via the IV and PICC routes, to include parameters such as, vector concentration, infusion volume, flow rate, and any anticipated differences in the biodistribution (BD) profile.

Sponsor Question 4:

For the immunogenicity assessment portion of the planned toxicology study, we plan to only perform in vivo anti-AAV9 capsid antibody testing. We will not conduct any additional immunogenicity assessments, such as ELISpot assays in WT animals or mouse antibodies against PCCA, since the mouse immune responses to the encoded transgene may not be informative towards human translation. Does the Agency agree?

CBER Response to Question 4:

We do not agree with your position to not evaluate potential immunogenicity to the AAV9-hPCCA transgene product in in your proposed GLP toxicology study. The murine immune response to the transgene product is an important factor in overall interpretation of the resulting data. Please include a plan to assess humoral and cellular immunogenicity to the PCCA transgene product in your GLP toxicology study protocol.

Sponsor Question 5a:

Regarding the proposed clinical synopsis (Section 2.3.1), does the Agency concur with the following:

a. The proposed study design?

CBER Response to Question 5.a:

No, we cannot agree with your current study design. You propose, page 29, an open-label Phase 1/2 dose-escalation study (three dose cohorts) of systemic AAV9-hPCCA gene therapy in up to 10 pediatric and adult PCCA patients, aged 2-18 years, with "clinically, biochemically, and/or molecularly confirmed PCCA genetic mutations that cause PA."

1. Given the inherent risks with gene therapy, in addition to clinical evidence, you must demonstrate that each subject has: (1) documented biochemical evidence of a deficiency in propionyl-CoA carboxylase (PCC) enzyme; and (2) pathogenic biallelic mutation(s) in the gene coding for this enzyme.

    a. Please explain whether you plan to use laboratory developed procedures for these critical eligibility decisions. We are concerned that these *in vitro* diagnostic (IVD) devices may represent significant risk devices under 21 CFR 812.3(m)(3) requiring an IDE.[1]

b. Regarding gene mutation and PCC assessments, please submit to your IND:

    i. Name, location, and CLIA certification of the diagnostic laboratory(ies)

    ii. Detailed test methodologies and/or SOP(s), and information regarding these *in vitro* devices and reagent qualification;

    iii. Validation level of the diagnostic test(s) for suitability (i.e., accuracy, sensitivity, specificity, reproducibility;

    iv. Information regarding QA/QC systems in place, and diagnostic test report sign-off procedure(s); and

    v. Risk assessments detailing the risk to the study population in case of false positive results leading to recruitment of study subjects who may not otherwise be eligible for enrollment in the study.

c. Regarding gene mutation assessments, please justify the mutations of the population you intend to include in your study.

d. Regarding PCC assessments, please:(1) clarify the threshold for PCC deficiency with justification; and (2) include PCC reference ranges with units of measure.

2. The branched chain acidurias, including PA, have three clinical presentations: (1) a severe neonatal-onset form with acute metabolic decompensation and neurological distress; (2) an acute, intermittent, late-onset form also with recurrent episodes of metabolic decompensation; and (3) a chronic, progressive form presenting as hypotonia, failure to thrive, and developmental delay. There are also various forms of disease, e.g., neurologic, hepatic, hematological, and immunological. Your briefing package does not provide enough information to determine whether the subpopulation(s) for your FIH study are appropriate. Since this first study is dose ranging, we recommend studying a homogeneous population of PA subjects to have interpretable results. When selecting your desired population, please ensure subjects have a favorable benefit-risk profile.

[1] See Guidance for Industry: In Vitro Companion Diagnostic Devices at 12 (explaining that "IVD companion diagnostic devices used to make treatment decisions in clinical trials of a therapeutic product generally will be considered investigational devices, unless employed for an intended use for which the device is already approved or cleared. **If used to make critical treatment decisions, such as patient selection, treatment assignment, or treatment arm, a diagnostic device generally will be considered a significant risk device** under21 CFR 812.3(m)(3) because it presents a potential for serious risk to the health, safety, or welfare of the subject, and **the sponsor of the diagnostic device will be required to comply with the investigational device exemption(IDE) regulations that address significant risk devices**.") (emphasis added) (accessed on 12 Jul 2021 at https://www.fda.gov/media/81309/download).

3. Your proposed study in children involves more than a minor increase over minimal risk, as stated above in our response to Question # 1, the additional safeguards under 21 CFR 50, Subpart D, and in particular 21 CFR 50.52 applies. Such clinical investigations must offer the PDB at all dose levels to pediatric subjects as demonstrated in animal models or adult subjects. For early phase human studies, it is especially important to enroll at least an initial cohort of adult subjects to obtain preliminary data on safety and feasibility, bioactivity, and preliminary efficacy to support enrollment of pediatric subjects. Because there are adults with the acute intermittent form of PA, please provide your rationale, with any available data, explaining why it would not be possible to achieve the primary study objectives by enrolling adult subjects.

4. You have proposed an open-label single-arm study without a comparator. While an open-label, single-arm study may be acceptable for an early-phase study, a concurrent control group will facilitate data interpretability with regard to safety, bioactivity and preliminary efficacy.

Due to rarity of PA and the substantial unmet medical need, we recommend that this early phase clinical trial should be adequately designed to demonstrate preliminary effectiveness to support marketing approval. To achieve this goal, we recommend that you modify your study design to employ a randomized control design, if feasible. If not feasible, please consider a prospective concurrent control with patients on standard of care who are not interested in gene therapy. If you ultimately determine that only a natural history control is feasible, please note that control patients should be as similar as possible to the population that will receive the experimental therapy with respect to disease sub-type, genotype, demographics, baseline functional status, and concomitant therapy with the exception of receiving the investigational product. Additionally, study observations should be performed using the same methodology and timing in the controls and the experimental subjects.[2]

Sponsor Question 5b:

a. The safety and sufficiency of a proposed initial human dose of [low dose] for FIH testing?

CBER Response to Question 5.b:

No, we do not agree. Please see our response to Question #2 above.

[2] For additional information, please see: E10 Choice of Control Group and Related Issues in Clinical Trials (May 2001) (accessed on Jul 12, 2021 at https://www.fda.gov/media/71349/download).

Sponsor Question 5c:

a. *The proposed three dose cohorts and dose escalation plan?*

CBER Response to Question 5.c:

We agree with the importance of dose exploration, however it is premature to comment on the acceptability of your proposed dose cohorts. Please see our response to Question #2 above.

Regarding dose escalation, you propose a staggering interval of at least 8 weeks between study participants. A DSMB review of available safety data will commence at the end of 8 weeks after dosing of each subject. The staggering interval between cohorts is unspecified.

Regarding the within-cohort staggering interval, it is premature to comment on its appropriateness because you did not provide justification. As a general rule, the staggering interval should be sufficient to monitor for acute and subacute adverse events. We also recommend that you specify a minimum period of safety follow-up for a cohort that will be reviewed prior to beginning the next dosing cohort. The observation periods within and between cohorts should be justified based on your preclinical experience and any other relevant scientific data.

Sponsor Question 5d:

b. *The FIH population with an age range of participants from 2 to 18 years?*

CBER Response to Question 5.d:

No, we do not agree. Please see our response to Question #1.

Sponsor Question 5e:

c. *The oral prednisone (1 mg/kg/day) for 4-6 weeks is appropriate to mitigate possible immune response to administration of AAV9-hPCCA?*

CBER Response to Question 5.e:

This may be an appropriate. Please justify the dose regimen for the population you wish to study. Please include a maximum non-weight-based dose.

Hyperammonemia is a characteristic feature of organic acidemias, particularly PA. We are concerned that prednisone administration may increase catabolism and potentially trigger a hyperammonemic crisis. In your protocol, please address

how you will monitor for hyperammonemic crises. Please include instructions to subjects and caregivers in the event of suspected hyperammonemia.

With prolonged steroids, subjects are at risk for suppression of the hypothalamic pituitary adrenal (HPA) axis. In your protocol, please include and justify: (1) the use of stress dose steroids; (2) processes to test HPA axis recovery; and (3) steroid tapering plan.

Sponsor Question 5f:

d. The primary and secondary endpoints in the proposed clinical study?

CBER Response to Question 5.f:

You propose a primary endpoint of the incidence of treatment-related adverse events, treatment-emergent adverse events, and serious adverse events for each cohort. You propose a secondary endpoint of absolute and percent change from baseline of [*biomarker 1 and biomarker 2*] at Week 52.

The primary safety endpoint is appropriate for a FIH study with gene therapy. However, given the rarity of this disease, we recommend that you also include an appropriate primary efficacy endpoint that reflects how a patient with PA will feel, function, or survive if they benefit from therapy with your product. Results from biomarker 1 are not sufficient as a primary efficacy endpoint because this is a surrogate marker. If you plan to use [*biomarker 1 and biomarker 2]* in your study, please provide detailed descriptions of these tests and address whether you will develop them as in vitro diagnostic devices.
For further information, please see FDA Guidance documents on in vitro companion diagnostics.[3]

Sponsor Question 5g:

e. The study duration and the proposed intervals for biomarker and preliminary efficacy testing?

CBER Response to Question 5.g:

You propose a study duration of 52 weeks. Subjects who complete the 12-month short-term study will roll into the long-term study, which will include interval assessment of safety and efficacy of AAV9-hPCCA for up to 5 years of follow-up.

The active phase of your study is likely insufficient given patients with PA may have a slowly progressive decline in function. We recommend that you consider a longer period of study to determine preliminary effectiveness of your product. In

---

[3] Guidance for Industry and Food and Drug Administration Staff: In Vitro Companion Diagnostic Devices (Aug 2014) (accessed on Jul 12, 2021 at https://www.fda.gov/media/81309/download); Guidance for Industry and Food and Drug Administration Staff: Principles for Codevelopment of an In Vitro Companion Diagnostic Device with a Therapeutic Product (Jul 2015) (accessed on Jul 12, 2021 at https://www.fda.gov/media/99030/download).

future submissions, please provide more details regarding your long-term follow-up (LTFU) plans. At a minimum, all subjects who receive a partial or full dose should be followed for collection of safety data for at least five years. Please note, because of the high-risk nature of gene therapy, you should not withdraw subjects from this study or the LTFU study unless the subject or legally authorized representative withdraws consent.

As a resource, FDA has a helpful guidance on long-term follow-up after gene therapy.[4]

Sponsor Question 6:

In light of recent safety signals related to the subacute complement cascade activation in recent trials of systemic high dose AAV9 mediated gene transfer in boys with DMD, does the Agency have any concerns and/or feedback regarding the proposed clinical protocol synopsis, risk mitigation strategy and AAV9-hPCCA development plan in the planned FIH study?

CBER Response to Question 6:

We are unable to comment on programs in clinical development. Please refer to the "Dear Health Care Professional Letter" issued March 18, 2021 by Novartis regarding onasemnogene abeparvovec—an AAV9 based therapy for spinal muscular atrophy.[5] Please note the warnings and precautions in the current label related to thrombotic microangiopathy (TMA) as information in this section may be applicable for your program.[6]

To ensure subjects safety, you may also consider incorporating the following suggestions in your protocol to mitigate complement mediated toxicity:

1. Design your product to minimize exposure to total viral particles. From the outset of your study, the percent of empty capsids delivered per dose should be as low as possible. Include a maximum ceiling for the total dose of viral particles administered.

2. Provide details on your Nab AAV9 assay, and justify the threshold for neutralizing antibodies as an eligibility criterion. It is possible that reactions from neutralizing antibodies (Nab) to the investigational product may contribute to the occurrence of TMA.

---

[4] FDA Guidance for Industry: Long Term Follow-Up After Administration of Human Gene Therapy Products (Jan 2020) (accessed on Jul 12, 2021 at https://www.fda.gov/media/113768/download).
[5] * See Dear Health Care Professional Letter (Mar 18, 2021) (accessed on Jul 12, 2021 at https://www.curesma.org/novartis-community-statement-zolgensma-safety-monitoring/).
[6] Zolgensma Prescribing Information (accessed on Jul 12, 2021 at https://www.novartis.us/sites/www.novartis.us/files/zolgensma.pdf).

3. Please do not administer gene therapy to subjects who have intercurrent infections.[7]

4. Please develop clear rules with justification for monitoring subjects for TMA and other safety signals related to the subacute complement cascade activation within your study. We recommend that you have a nephrologist, experienced in the management of TMA, to consult with Investigators on all cases.

5. Provide laboratory and clinical parameters with justification that will trigger treatment with eculizumab. Follow the labeled instructions for dosing and monitoring subjects on eculizumab. There is a boxed warning for life-threatening and fatal meningococcal infections after treatment with eculizumab. Please consider vaccinating subjects against meningococcal infections well before gene therapy administration, e.g., 6 weeks.

6. It may be helpful if your DSMB includes clinicians with expertise in gene therapy and a physician who has expertise in diagnosis, treatment, and clinical consequences of TMA. Please consider including a physician-expert in TMA as a member of your DSMB.

Sponsor Question 7:

Based on the presented POC animal studies and supporting rationale, outlined under Section 2.5, Planning for Expedited Pathways, does the Agency agree with our proposal to submit a Fast Track (FT) designation request at time of IND submission?

CBER Response to Question 7:

It is premature to comment on the appropriateness of a Fast Track designation. If you wish to submit a Fast Track Designation Request, please refer to FDA's Guidance on Expedited Programs for Serious Conditions.[8]

Sponsor Question 8:

We are proposing submission of a Regenerative Medicine Advanced Therapy Designation (RMAT) designation request within the first two months of the Phase 1/2 FIH study utilizing response biomarker data (Section 2.5). Does the Agency agree with the proposed timing of RMAT designation request?

CBER Response to Question 8:

It is premature to comment on the appropriateness of an RMAT designation request. Please see our Guidance on Expedited Programs for Regenerative Medicine

[7] Chand D, et. al., Thrombotic Microangiopathy Following Onasemnogene Abeparvovec for Spinal Muscular Atrophy: A Case Series, J Pediatr. 2021 Apr;231:265-268 (describing an overview of thrombotic microangiopathy reports for SMA patients treated with onasemnogene abeparvovec).

[8] FDA Guidance for Industry: Expedited Programs for Serious Conditions—Drugs and Biologics (May 2014) (accessed on Jul 12, 2021 at https://www.fda.gov/media/86377/download).

Therapies for Serious Conditions.[9] Please note that RMAT designation requires preliminary clinical evidence that the product will meet an unmet medical need. Evidence from biomarkers is not sufficient for the purpose of RMAT designation.

Sponsor Question 9:

As per our understanding from our meeting with the Agency on July 2, 2019 and FDA Guidance for Industry, "Preclinical Assessment of Investigational Cellular and Gene Therapy Products", November 2013, is the Agency still in agreement that the planned biodistribution studies for AAV9-hPCCA would be sufficient to support preclinical development programs for subsequent gene targets, utilizing our established AAV9 delivery capsid platform (i.e., different gene target and intended population)? We acknowledge that the Agency would like for us to submit a separate IND for each of the four rare disease indications discussed above.

CBER Response to Question 9:

We tentatively agree that the resulting BD data for AAV9-hPCCA from a well-designed study can be leveraged to support a BD platform for the same AAV9 delivery capsid with a different gene target. However: 1) the BD data for AAV9-hPCCA will need to be sufficient to support the proposed FIH clinical trial; 2) the same route of administration (IV injection) should be used; and 3) supplemental data for transgene expression in tissues positive for vector presence may be needed to support clinical trials with AAV9 vector products that include different promoters, target tissues, etc.

Sponsor Question 10:

Does the Agency agree that the planned preclinical anti-drug antibody (ADA) measured against the AAV9 capsid in the AAV9-hPCCA toxicology study, would be sufficient to support subsequent planned gene therapy development programs utilizing the same AAV9 capsid under a different IND (i.e., different gene target and intended population)?

CBER Response to Question 10:

We tentatively agree that ADA data for AAV9-hPCCA can be leveraged to support other products using the same AAV9 vector construct. However, please see our comment to your Question #4 regarding evaluation of potential immunogenicity to the expressed transgene product.

Sponsor Question 11:

Can the Agency provide feedback on whether a drug master file/s (DMF) would be recommended for the PaVe-GT project?

[9] Expedited Programs for Regenerative Medicine Therapies for Serious Conditions (accessed on Jul 12 , 2021 at https://www.fda.gov/media/120267/download).

CBER Response to Question 11:

Please note that as the IND sponsor you are ultimately responsible for providing all the CMC information necessary to assess product safety for the planned Phase 1 trial either as part of the original submission or via a cross-referenced Master File (MF) or IND. For our comprehensive recommendations on the CMC content of an IND to initiate clinical trials, please refer to the FDA guidance "Chemistry, Manufacturing, and Control (CMC) Information for Human Gene Therapy Investigational New Drug Applications (INDs)", dated January 2020, (https://www.fda.gov/media/113760/download). Please also note the following:

1. If you intend to reference CMC information in your IND that was previously submitted to the Agency under another IND or MF, then you should clearly specify (preferably in a tabular format) the information to be referenced, including the nature of the information (e.g., reagents, testing, manufacturing, etc.), file name, reference number, eCTD module, and page number where the information can be found. This information should also be clearly stated in the Letter of Authorization (LOA) provided by the cross-referenced IND sponsor or MF holder.

2. A MF can allow you (the sponsor) to incorporate, by reference, information contained in the MF without the MF holder [e.g., the Contract Manufacturing Organization CMO)] having to disclose proprietary information. The FDA will not discuss the details of your IND submission with the MF holder or IND sponsor without written authorization to do so.

3. If the CMO elects to use a MF to document CMC information that will be needed for IND review, we recommend communicating with them prior to submission of your IND to the FDA. Particularly, we recommend submitting the MF well in advance of the IND to allow adequate time for the agency to review the CMC information (that will support the IND) within the 30-day review cycle (for the IND).

4. In the MF, product-specific information (e.g., testing and/or manufacturing details, stability) should be submitted under distinct Sections (for each product), e.g., Section 3.2.S – DS for Product A (in this case, AAV9-hPCCA), Section 3.2.S DS for Product B – Product B, Section 3.2.S DS for Product C and so on.

5. Please note that technical contents that describe Drug Substance (DS)/Drug Product (DP) manufacture (including reagents, manufacturing processes, testing, and manufacturing facilities) would be submitted as a type 2 Master File (MF2). For additional guidance on the type of facility-related information that should be submitted in the MF2 submission (that you would otherwise submit in the IND), please refer to Sections V.A.1 and V.A.2, and Section V.5.C.1 (Appendices) in the following guidance document: Chemistry, Manufacturing, and Control (CMC) Information for Human Gene Therapy Investigational New Drug Applications (INDs), 2020. Chemistry, Manufacturing, and Control (CMC) Information for Human Gene Therapy Investigational New Drug Applications (INDs) | FDA.

Please note that additional information for the manufacturing facilities can also be separately submitted in a type 5 MF (MF5). In which case, this MF5 should also be cross-referenced in your IND submission.

6. Please be advised that the product(s) you are proposing to study under an IND are considered biologics regulated by the Center for Biologics Evaluation and Research (CBER); therefore, it is critical that any MF you and your CMO elect to utilize must be submitted to CBER's electronic submission gateway (ESG). Please find additional information here: https://www.fda.gov/vaccines-blood-biologics/development-approval-process-cber/master-files-cber-regulated-products.

7. Please be advised that MFs should be submitted through CBER's ESG in eCTD format instead of PDF format.

8. Please be aware that an applicant cannot cross-reference MFs for the information on DS/DP/DS intermediate manufacture to support a Biologics Licensure Application (BLA). Information on product manufacturing and testing must be provided in the BLA.

9. Please note that MFs are submitted solely at the discretion of their holders (e.g., CMOs) and are not required by statute or regulation.

10. Detailed information on the contents and format of MFs can be found in the FDA guidance "Drug Master Files", dated November 2020, (https://www.fda.gov/media/131861/download).

We agree that Pharmacology/Toxicology data used to support the PaVe-GT project and intended to be used to support multiple IND submissions, can be submitted under a Drug Master File.

Additional CBER Comments:

General Considerations

1. We recommend that you request a pre-IND meeting with CBER/OTAT when ready, to obtain formal nonbinding comments regarding your product development plan from the three CBER/OTAT review disciplines, consisting of product manufacturing (CMC), pharmacology/toxicology (P/T), and clinical. Please be advised that you should consider and address all recommendations provided in these INTERACT comments when you submit a pre-IND meeting package.

2. We refer you to *OTAT Learn*, a series of online presentations provided by the Office of Tissues and Advanced Therapies (OTAT) which address important topics in the development of products regulated by OTAT. You may find some of

these presentations useful in your preparation of regulatory submissions and briefing materials for meetings with FDA. *OTAT Learn* is available at https://www.fda.gov/biologicsbloodvaccines/newsevents/ucm232821.htm.

3. FDA has published the following guidance documents that may be helpful when drafting and revising your clinical protocols:

   a. Guidance for Industry: Considerations for the Design of Early-Phase Clinical Trials of Cellular and Gene Therapy Products[10]; and

   b. Rare Diseases: Common Issues in Drug Development.[11]

4. Drug development is a collaboration of diverse stakeholders, such as the pharmaceutical industry, academicians, patients, advocacy groups, and regulators. Transparency in the regulatory process can facilitate that collaboration. Therefore, please consider inviting one or more patients, representatives of patient advocacy groups, and/or investigators to your future interactions such as meetings or teleconferences with the FDA. We recognize that, depending on the nature of the topics under discussion and the time available, it may not be feasible for all such stakeholders to actively participate in the discussion. However, listening to the proceedings of such meetings can provide a valuable opportunity for patient stakeholders to contribute to drug development.

5. In accordance with provisions of the 21st Century Cures Act, FDA intends to utilize patient experience data and related information in regulatory review and decision-making. Patient experience data provide information about the impact of a medical condition or a therapy on a patient's life, and information about the patient's preferences for treatment. Data may be collected by any persons (including patients, family members and patients' caregivers, patient advocacy organizations, disease research foundations, researchers, and drug manufacturers). We encourage you to incorporate patient experience data as supportive information to guide medical product development. Additional information can be found at: https://www.fda.gov/downloads/Drugs/GuidanceComplianceRegulatoryInformation/Guidances/UCM610442.pdf

---

[10] Guidance for Industry: Considerations for the Design of Early-Phase Clinical Trials of Cellular and Gene Therapy Products (accessed on Jul 12, 2021 at https://www.fda.gov/media/106369/download).

[11] Guidance for Industry: Rare Diseases: Common Issues in Drug Development Guidance for Industry (accessed on Jul 12, 2021 at https://www.fda.gov/media/119757/download).

Chemistry, Manufacturing, and Control

Please consider the following recommendations for the CMC information that should be submitted to the IND:

1. Please note that plasmids used in the manufacturing of AAV vectors are considered critical starting materials. In your IND submission, please include the following:

    a. Certificates of Analysis (COAs) for the plasmids used in the manufacturing process that includes the grade of plasmid. Please note that research grade plasmids should not be used for the manufacture of AAV gene therapy vectors used in clinical studies.

    b. Annotated maps for each plasmid indicating the origin, function, and derivation of each genetic component (i.e., promoters, introns, known coding sequences, polyadenylation signals, and untranslated regions).

    c. Summary reports of sequence analysis for each plasmid used in the manufacture of the product, including sequence analysis of all elements in the plasmid that are key to vector production. The summary should include an explanation of any discrepancies with the expected/reference sequence.

    d. A description of the procedures put in place by the manufacturer of the plasmids to prevent cross-contamination from other plasmid lots made at the facility in prior campaigns (i.e., segregation, tracking, and changeover systems for manufacturing of the plasmid DNA lots).

    e. A complete list of raw materials and manufacturing equipment used to make the plasmids (please denote which materials are single-use and which are reused).

    f. A complete list of the animal-derived products used to make the plasmids.

    g. A summary report for cleaning validation studies for any equipment or materials that are not single use.

    h. Description of the procedures/testing put in place at the GMP product manufacturing facility to qualify the incoming plasmids as critical starting material.

2. Regarding the assay for measurement of vector strength/concentration (commonly referred to as the vector genome titer assay, e.g., qPCR or ddPCR) used for dose calculation, please note the following:

    a. The assay must be qualified (suitable for the intended purpose) prior to Phase

1 clinical studies. Failure to submit adequate information supporting assay qualification will result in your IND being placed on clinical hold.

b. Please collect assay qualification data for the product under the study and include appropriate reference standards of consistent quality and nature, (e.g., linear DNA vs circular plasmids as standards used in the qPCR assay) and product-specific controls/reference material.

c. In the IND, please provide a detailed protocol for the qualification study or the SOP used to qualify your assay, including information on the reference standards, controls, and assay optimization.

d. Please provide the study report with data documenting assay qualification, including accuracy, precision (inter-assay and intra-assay precision), specificity, range, and linearity. We recommend that the precision of the qualified/validated assay be less than 15% coefficient of variation (CV).

e. Please describe any deviations that occurred during the qualification study.

f. Please plan to validate the assay prior to the conduct of clinical studies that will assess product efficacy for licensure.

g. For additional recommendations on assay qualification/validation, please refer to ICH Q2R1 "Validation of Analytical Procedures: Text and Methodology" (https://database.ich.org/sites/default/files/Q2%28R1%29%20Guideline.pdf); and FDA guidance "Analytical Procedures and Methods Validation for Drugs and Biologics", dated July 2015, (https://www.fda.gov/media/87801/download).

3. To ensure consistent dosing between clinical and preclinical studies, we recommend using the same qualified assay for calculating the vector strength/titer (i.e., vector genome concentration) of preclinical and clinical lots. In instances where this is not possible, you should plan to conduct a bridging study that will assess the vector strength/titer of the product of the preclinical lot(s) and clinical lot (planned for Phase 1 study) in a side-by-side manner, with the assay that will be used for measuring vector strength/titer of the clinical lots. Please plan to demonstrate a tight correlation between the two different assays using multiple product lots (development, engineering, preclinical lots, etc.). This may require retaining sufficient quantities of the product lots.

4. In your pre-IND briefing package, please provide your proposed lot release testing plans for DS and DP. We recommend that you set specifications for process-related impurities (process residuals such as host cell protein, host cell DNA, plasmid DNA. nuclease, bovine serum albumin, etc.) and product-related impurities (e.g., empty-to-full capsid [E/F] ratio, aggregates etc.) based on your experience from manufacturing of development lots, preclinical lots, and clinical

lots of AAV9-hPCCA, as well as data from manufacturing other AAV vectors using the same manufacturing platform, if available.

5. You should also submit a tabular listing of all the in-process testing planned for your manufacturing platform and the 'go-no go' criteria/limits set for further processing.

6. Because you are developing products for rare diseases, it is likely that your clinical plan will involve a small trial to support a marketing application, which may require only a few clinical lots. Considering that, we recommend that you develop and implement a quantitative and biologically relevant potency assay for release testing and stability testing of clinical lots. We have the following recommendations on potency assay development:

   a. Potency assay should be qualified before initiation of clinical trials intended to provide primary evidence of effectiveness to support a marketing application and validated prior to submission of a marketing application (BLA).

   b. You may consider developing a matrix of assays to measure the product potency, considering the mechanism of action of the product. In this approach you will measure different aspects of the biological activity of the product (e.g., infectivity, transgene expression, enzymatic activity) as part of the potency matrix.

   c. We recommend collecting potency data early in clinical development.

   d. In our experience, potency testing can involve large volumes of sample material; therefore, we recommend that you plan for adequate number of retains from each lot (IND-enabling lots and clinical lots). This can be useful for future testing, assay development, and comparability studies, etc.

   e. For additional guidance and relevant information on potency assay development, please refer to the following FDA/CBER guidance document "Guidance for Industry: Potency Tests for Cellular and Gene Therapy Products", dated January 2011 (https://www.fda.gov/regulatory-information/search-fda-guidance-documents/potency-tests-cellular-and-gene-therapy-products).

7. Considering your plan to develop a platform approach, we recommend that you develop sensitive and precise assays as part of heightened product characterization to evaluate product quality attributes such as the empty-to-full capsid (E/F) ratio, vector particle aggregation, post-translation modifications of the capsid, etc., using more than one approach (assay). You should develop these assays at the early stages of the product development to identify those suitable for lot release testing and product characterization. Please note that all assays used in the analytical release testing plan should be qualified (suitable for

the intended purpose). The qualification data (for assay performance: accuracy, precision, linearity, sensitivity and specificity, as applicable to each assay) should be submitted in the IND.

8. Please optimize the formulation of final DP taking into consideration product stability during manufacturing (in process stability for example during hold steps), under the conditions of storage, shipping and handling in the clinic (including during administration in the device). Please provide the rationale for the final formulation with supporting data from formulation development studies.

9. Please submit (in your IND) a stability study protocol and any available data supporting the stability of the Drug Substance and Drug Product for the duration of storage. Please note the following recommendations:

    a. Please note that stability protocols should include, but are not limited to, an analysis for product potency, evaluation of product quality, and confirmation of sterility. Please note that, in lieu of sterility testing beyond T=0, you may consider container closure integrity testing according to FDA's 2008 guidance "Container and Closure System Integrity Testing in Lieu of Sterility Testing as a Component of the Stability Protocol for Sterile Products" (https://www.fda.gov/media/76338/download).

    b. For description of each product attribute measured in the stability plan, please include the test method, sampling time points (there should be a zero-time point), and acceptance criteria. Please note that under 21 CFR 312.23(a)(7)(ii), you must conduct stability testing in all phases of the IND, to demonstrate that the product is within acceptable chemical and physical limits for the planned duration of the proposed clinical investigation.

    c. Please consider placing preclinical/engineering lots and all clinical lots on the stability plan.

    d. In addition to the real-time stability under normal storage conditions, please also assess the stability of the DP under accelerated storage and forced degradation conditions to allow for the identification of stability-indicating assays early in the clinical development.

    e. For recommendations on stability testing, please refer to ICH Q5C: "Quality of Biotechnological Products: Stability Testing of Biotechnological/Biological Products," (https://www.fda.gov/media/71441/download), ICH Guideline Q1A(R2): "Stability Testing of New Drugs and Products" (https://www.fda.gov/media/71707/download), and ICH Guideline Q1E: "Evaluation of Stability Data" (https://www.fda.gov/media/71722/download).

10. Please establish a product specific reference standard material for assays that measure critical quality attributes such as vector genome titer, infectious unit

titer, E/F capsid ratio, etc. Please note:

a. In the IND/MF, please provide a detailed description of how the reference standard material was derived, manufactured and qualified.

b. Please test the reference material (lot) according to the product release testing plan and product characterization plan. Please submit the COA for the reference lot.

c. Please monitor the stability of this reference lot.

d. Please provide your justification for why this reference lot is relevant and acceptable.

11. Please collect device compatibility data for AAV9-hPCCA showing the stability of the product in the intended clinical formulation and clinical delivery device. Please ensure that the product lot used in the compatibility study is manufactured and formulated in a manner comparable to that for the clinical lots. The supporting manufacturing, qualification and testing information for the product lot used in the compatibility study should be submitted in the IND. We have the following additional comments regarding the device compatibility study:

    a. Please assess the amount (vector genomes) and activity (infectious units or potency) of the product following exposure to the clinical delivery device.

    b. Please be aware that the study should include tests conducted over the planned dose-range and should take into account the expected time between thaw of the product and infusion. Please perform device compatibility testing for the product under conditions that mimic the clinical scenario (i.e., hold time, formulation/concentration, temperature, presence of contrast agent, etc.); the study design should consider the worst-case scenario (e.g., low product concentration, maximum hold time, time and temperature excursions).

    c. The data should support the post-thaw product handling instructions provided in the “Instruction to Pharmacy” or “Pharmacy Manual” document that is supplied with the product to all the clinical sites.

    d. Failure to submit sufficient information supporting product (vector) stability in the delivery device will result in your IND being placed on clinical hold.

12. If you intend to use an assay/test to make treatment decisions (e.g., for the selection of subjects using a screening assay), such an assay may be regulated as a companion diagnostic, in which case, we encourage you to request a Pre-Submission meeting with Office of In Vitro Diagnostics and Radiological Health within CDRH's Office of Product Evaluation and Quality (OPEQ) to get advice on

the development plan for this assay. Please note that participation of the OTAT review team (for the IND) can be requested at that meeting and would be helpful in ensuring contemporaneous development of both the therapeutic and the diagnostic device/assay.

Furthermore, we encourage you to submit the same information (as that submitted with the Pre-Submission package to CDRH) as an amendment to the IND to document assay development.

For additional guidance, please refer to the following guidance documents:

a. The 2014 FDA guidance document on "In Vitro Companion Diagnostic Devices" (https://www.fda.gov/media/81309/download)

b. The 2021 FDA guidance document on "Requests for Feedback and Meetings for Medical Device Submissions: The Q-Submission Program" (https://www.fda.gov/media/114034/download)

Pharmacology/Toxicology

13. For a comprehensive summary regarding the preclinical assessment of cell and gene therapy products, we refer you to the document titled, *Guidance for Industry: Preclinical Assessment of Investigational Cellular and Gene Therapy Products* (November 2013), available at: https://www.fda.gov/regulatory-information/search-fda-guidance-documents/preclinical-assessment-investigational-cellular-and-gene-therapy-products.

14. The preclinical program for any investigational product should be individualized with respect to scope, complexity, and overall design to maximize the contribution and predictive value of the resulting data for clinical safety and therapeutic activity. As recommended in Section III.B.8 of the *Guidance for Industry: Preclinical Assessment of Investigational Cellular and Gene Therapy Products* we encourage you to explore opportunities for reducing, refining, and replacing animal use in your preclinical program. For example, it may be appropriate to use *in vitro* or *in silico* testing to complement or replace animal studies. We encourage you to submit proposals and justify any potential alternative approaches.

Clinical

15. You propose to include neurocognitive assessments at baseline and 52 weeks. These assessments are performed with Clinical Outcomes Assessments (COA) tools, e.g., Bayley, Vineland. For all COA tools that are intended to support regulatory decision-making and labeling claims, we recommend that you provide the following for FDA review and comment prior to initiating your study:

a. Conceptual framework of the instrument;

b. Evidence of content validity obtained for the tool, e.g., qualitative research with caregivers, obtained for this specific context of use;

c. Exact copy of the instrument as it will be administered during the clinical trial and any training materials and user manuals;

d. Proposed scoring algorithm(s) with rationale for any weighting of items or response options in the domain scoring and corresponding information on how the instrument's scores will be analyzed as part of an endpoint;

e. Plans for, and results from, evaluation of the psychometric properties and performance of the instrument (i.e., reliability, validity, and ability to detect change) after content validity has been established;

f. Pre-specified plans for handling missing data; and

g. *A priori* thresholds (or range of thresholds) representing clinically meaningful within-patient improvement in instrument's scores.

When we have this information, we may be able to provide further feedback on whether the tool is an acceptable efficacy endpoint. As a resource on COAs, please see FDA's Guidance for Industry: Patient-Reported Outcome Measures: Use in Medical Product Development to Support Labeling Claims (March 2009).[12]

16. You state, page 11, that "[s]tandard of care includes life-long dietary protein restriction, supplementation with L-carnitine and supportive treatment." We are concerned that "dietary changes can affect efficacy results and pose significant interpretability challenges, particularly when the clinical trial design did not anticipate or appropriately account for the confounding effect of diet." In drafting your protocol with regard to diet, please see FDA's guidance on optimizing, standardizing, and maintaining diet stability in clinical trials.[13]

17. Your study has Data and Safety Monitoring Board to review safety data and make recommendations on pausing the study or modifying the protocol. Please provide the DSMB's Charter for our review when you submit your IND.

[12] Guidance for Industry: Patient-Reported Outcome Measures: Use in Medical Product Development to Support Labeling Claims (Dec 2009) (accessed on Jul 12, 2021 at https://www.fda.gov/media/77832/download).

[13] Guidance for Industry: Inborn Errors of Metabolism That Use Dietary Management: Considerations for Optimizing and Standardizing Diet in Clinical Trials for Drug Product Development (Jul 2018) (accessed on Jul 12, 2021 at https://www.fda.gov/media/114764/download).

At a minimum, the Charter should include the following information:

a. Qualifications of DSMB members:

b. Roles and responsibilities of members;

c. Detailed explanation for decision-making;

d. Meeting schedule, i.e., time interval from detection of serious adverse event to DSMB notification, e.g., 24 hours, and adjudication, e.g., 72 hours;

e. DSMB discussions should take place at a live meeting (telephone, videoconference) to ensure views of all members can be heard collectively. Prior to the meeting, however, members should independently determine their recommendation(s), and disclose them to other members, to diminish the possibility that a member could dominate the discussion and overly influence the decision;

f. Voting rules, e.g., whether unanimity or two-thirds majority is required for decision-making;

g. Written rules on procedures to ensure minimization of bias, such as maintaining confidentiality of the interim data;
h. Documentation of decisions, e.g., where summary of adjudication discussions and recommendations are memorialized, documentation of all communications with sponsor;

i. Training of DSMB members

j. Kick-off meeting to ensure that all members understand the protocol and decision-making rules prior to first subject enrollment;

k. Ongoing assessment of DSMB members for conflicts of interest, including concurrent service on other DSMBs of related or competing products; and

l. Replacement of members upon resignation or conflict of interest.

As a helpful resource, please see FDA's Guidance for the establishment and operation of data monitoring committees.[14]

18. In addition to the protocol and Informed consent Document, we recommend that you include the following documents in your IND submission.
    a. LTFU protocol;

[14] See Guidance for Clinical Trial Sponsors: Establishment and Operation of Clinical Trial Data Monitoring Committees (2006) (accessed on Jul 12, 2021 at https://www.fda.gov/media/75398/download).

b. LTFU Informed Consent Document;

c. Investigator Brochure as required by 21 CFR 312.55(a);

d. Committee Charters, e.g., DSMB Charter;

e. Pharmacy Manual inclusive of dose calculation worksheets;

f. Administration Manual, if applicable;

g. Any documents with an internal reference in the protocol, e.g., study manual, study SOPs, etc.

NCATS executive summary of teleconference discussion with FDA

After reviewing the written responses from the FDA, the PaVe-GT team decided to discuss Question 4 and Question 5 (a,d, and e) at the teleconference as these needed further clarity. For the other responses, we agreed with FDA's feedback. We communicated to the FDA in advance of the teleconference our intent to discuss the above questions along with supporting information. The questions and FDA written responses were discussed at length with input from both sides.
Stated below is NCATS executive from the INTERACT teleconference discussion held on July 14 2021.

1) **Immunogenicity assessments in animal studies (related to Question 4):** FDA was in alignment with our plan to evaluate the humoral immunogenicity of the capsid and the transgene and the cellular immunogenicity of the capsid alone in GLP toxicology study is reasonable. Additional advice was to save blood samples from the murine toxicology study (if feasible), to evaluate the cellular immune response of the transgene in the event there are there are differences in the clinical study toxicology findings that require further investigation.

2) **FIH Population (related to Question 5d)** – FDA requested for additional information in the pre-IND package as to why adults would be an infeasible FIH population, and why pediatric patients would be better suited; animal data could help support the proposal. The justification should also align with the Prospective Direct Benefit (PDB) regulatory requirement. Also, it was suggested to have a more homogenous pediatric population by narrowing the proposed 2-18 age range to older pediatric patients.

3) **Diagnostic tests (related to Question 5a, FDA response 1)**– The FDA initially recommended developing the PCC enzymatic activity assay as a companion diagnostic and to refer to the FDA guidance on in vitro diagnostics. However, after the discussion, it was suggested that the Sponsor should provide additional justification in the next submission as to why the enzymatic assay is not the best approach for diagnosis, along with including the current standard of care, clinical and molecular testing.
